# Physiological Traits Associated with Wheat Yield Potential and Performance under Water-Stress in a Mediterranean Environment

**DOI:** 10.3389/fpls.2016.00987

**Published:** 2016-07-07

**Authors:** Alejandro del Pozo, Alejandra Yáñez, Iván A. Matus, Gerardo Tapia, Dalma Castillo, Laura Sanchez-Jardón, José L. Araus

**Affiliations:** ^1^Programa de Investigación de Excelencia Interdisciplinaria, Adaptación de la Agricultura al Cambio Climático (A2C2), Facultad de Ciencias Agrarias, Centro de Mejoramiento Genético y Fenómica Vegetal, Universidad de TalcaTalca, Chile; ^2^Departamento de Ciencias Agrarias, Facultad de Ciencias Agrarias y Forestales, Universidad Católica del MauleCuricó, Chile; ^3^Centro Regional Investigación Quilamapu, Instituto de Investigaciones AgropecuariasChillán, Chile; ^4^Centro Universitario de la Patagonia, Universidad de MagallanesCoyhiaque, Chile; ^5^Unitat de Fisiologia Vegetal, Facultat de Biologia, Universitat de BarcelonaBarcelona, Spain

**Keywords:** carbohydrate, carbon isotope discrimination, chlorophyll, drought, stem reserves

## Abstract

Different physiological traits have been proposed as key traits associated with yield potential as well as performance under water stress. The aim of this paper is to examine the genotypic variability of leaf chlorophyll, stem water-soluble carbohydrate content and carbon isotope discrimination (Δ^13^C), and their relationship with grain yield (GY) and other agronomical traits, under contrasting water conditions in a Mediterranean environment. The study was performed on a large collection of 384 wheat genotypes grown under water stress (WS, rainfed), mild water stress (MWS, deficit irrigation), and full irrigation (FI). The average GY of two growing seasons was 2.4, 4.8, and 8.9 Mg ha^−1^ under WS, MWS, and FI, respectively. Chlorophyll content at anthesis was positively correlated with GY (except under FI in 2011) and the agronomical components kernels per spike (KS) and thousand kernel weight (TKW). The WSC content at anthesis (WSCCa) was negatively correlated with spikes per square meter (SM2), but positively correlated with KS and TKW under WS and FI conditions. As a consequence, the relationships between WSCCa with GY were low or not significant. Therefore, selecting for high stem WSC would not necessary lead to genotypes of GY potential. The relationship between Δ^13^C and GY was positive under FI and MWS but negative under severe WS (in 2011), indicating higher water use under yield potential and MWS conditions.

## Introduction

Since the Green Revolution the yields of wheat and other cereals have increased considerably in many regions of the world, including Chile (Calderini and Slafer, 1998; Engler and del Pozo, 2013; del Pozo et al., 2014), as a result of genetic improvement and better agronomic practices. The yield potential, i.e., the yield achieved when the best available technology is used, has also increased almost linearly since the sixties, particularly in more favorable environments where soil water availability is not limited (Zhou et al., 2007; Fischer and Edmeades, 2010; Matus et al., 2012; del Pozo et al., 2014). Yield under water-limiting conditions, such those of the rainfed Mediterranean environments, has also increased during the past decades (Sánchez-García et al., 2013). Notwithstanding the possible need for phenological adjustment (earliness) a higher yield potential may also translate into a higher performance under water stress (Nouri et al., 2011; Hawkesford et al., 2013). However, the potential yield and water-limited yield of wheat needs to continue increasing in order to cope with future demand for food, which is a consequence of the growing population and changes in social habits (Fischer, 2007; Hawkesford et al., 2013), and also to reduce the negative impacts on crop productivity of global climate change (Lobell et al., 2008; Lobell and Gourdji, 2012).

The increase, in the yield potential and stress adaptation of wheat has been attained mainly through empirical selection for grain yield (GY). However, there is evidence that phenotyping using physiological traits, as a complement to agronomic traits, may help in identifying selectable features that accelerate breeding for yield potential and performance under drought (Araus et al., 2002, 2008; Fischer, 2007; Foulkes et al., 2007; Cattivelli et al., 2008; Fleury et al., 2010). The increases in yield potential of wheat since the sixties have been both positively correlated with shoot dry matter and harvest index (HI); the latter also being positively associated with water-soluble carbohydrate (WSC) content of stems at anthesis (Foulkes et al., 2007). Under water limiting conditions, various physiological process and traits have been associated with GY (e.g., Araus et al., 2002, 2008; Condon et al., 2004; Reynolds et al., 2006; Tambussi et al., 2007). Among them are traits related to pre-anthesis accumulation of WSC in stems and its further use during grain filling (Ehdaie et al., 2006a,b; Reynolds et al., 2006), delays in senescence during grain filling assessed via changes in leaf color (Lopes and Reynolds, 2012), and those related to water use efficiency, in particular carbon isotope discrimination (Δ^13^C) in kernels (Richards et al., 2002; Araus et al., 2003, 2008).

WSCs are accumulated in stems prior to anthesis and then are remobilized to the grain during the grain-filling period (Blum, 1998; Bingham et al., 2007). Under water limiting conditions, where canopy photosynthesis is inhibited, the contribution of stem carbohydrate to grain growth could be very significant (Ehdaie et al., 2006a,b; Reynolds et al., 2006). Both spring and winter wheat lines have been shown to vary significantly for WSC concentration and WSC content in stems around anthesis (Ruuska et al., 2006; Foulkes et al., 2007; Yang et al., 2007), whereas positive correlations have been observed between accumulated WSC at anthesis and GY in winter wheat genotypes (Foulkes et al., 2007), as well as with kernel weight in recombinant inbred lines (RILs) from the Seri/Babax population (Dreccer et al., 2009). However, stem WSC concentrations can be negatively correlated with stem number m^−2^ (Dreccer et al., 2013).

Drought increases senescence, by accelerating chlorophyll degradation, leading to a decrease in leaf area and canopy photosynthesis. There is evidence that stay-green phenotypes with delayed leaf senescence can improve their performance under drought conditions (Rivero et al., 2007; Lopes and Reynolds, 2012).

Δ^13^C can be used as a selection criterion for high water use efficiency (Condon et al., 2004; Richards, 2006), but also can provide an indirect determination of the effective water used by the crop (Araus et al., 2002, 2008; Blum, 2009). In fact, kernel Δ^13^C can be positively or negatively correlated with GY depending on soil water availability. Indeed, under moderate stress to well-watered Mediterranean conditions Δ^13^C has been reported to be positively correlated with GY in wheat (Araus et al., 2003, 2008 for wheat) and barley (Acevedo et al., 1997; Voltas et al., 1999; del Pozo et al., 2012), whereas the opposite trend has been reported under severe drought conditions (but see Araus et al., 1998).

In this study we investigated the genotypic variability of flag leaf chlorophyll content (measured with a portable leaf meter), stem WSC accumulation at anthesis and the Δ^13^C of mature kernels, as well as the relationship of these traits with GY and its agronomical components, in spring bread wheat under contrasting water conditions in a Mediterranean environment. It is hypothesized that within a large set (384 genotypes) cultivars and advanced lines of spring bread wheat there is high genotypic variability for agronomic and physiological traits. In addition, the yield performance of genotypes under drought conditions is associated with stem WSC accumulation, delayed leaf senescence, and carbon discrimination in grains.

## Materials and methods

### Plant material and growing conditions

A collection of 384 cultivars and advanced semidwarf lines of spring bread wheat (*Triticum aestivu*m L.), including 153 lines from the wheat breeding program of the Instituto de Investigaciones Agropecuarias (INIA) in Chile, 53 from the International Wheat and Maize Improvement Centre (CIMMYT) that were previously selected for adaptiveness to Chilean environments (these lines share common ancestors with the INIA-Chile breeding program), and 178 lines from INIA in Uruguay (Table S1). The objective with this set of lines was to create a germplasm base to breed for drier areas in Chile and subsequently other countries within the projects involved.

This large set of genotypes was evaluated in two Mediterranean sites of Chile: Cauquenes (35°58′ S, 72°17′ W; 177 m.a.s.l.) under the water stress (WS) typical of the rainfed at this site, and Santa Rosa (36°32′ S, 71°55′ W; 220 m.a.s.l.) under full irrigation (FI) and moderate water stress (MWS) conditions achieved through support irrigation. Trials were assayed during two consecutive (2011 and 2012) crop seasons, except for the MWS trial, which was only set up during 2011. Cauquenes corresponds to the Mediterranean drought-prone area of Chile; the average annual temperature is 14.7°C, the minimum average is 4.7°C (July) and the maximum is 27°C (January). The evapotranspiration is 1200 mm (del Pozo and del Canto, 1999) and the annual precipitation was 410 and 600 mm in 2011 and 2012, respectively. Santa Rosa corresponds to a high yielding area; the average annual temperature in this region is 13.0°C, the minimum average is 3.0°C (July) and the maximum is 28.6°C (January; del Pozo and del Canto, 1999). The annual precipitation was 736 and 806 mm, in 2011 and 2012, respectively.

The experimental design was an α-lattice with 20 incomplete blocks per replicate, each block containing 20 genotypes. In each replicate two cultivars (Don Alberto and Carpintero) were included eight times. Two replicates per genotypes were used, except at Cauquenes and Santa Rosa SI in 2011 where a single replicate was established. Plots consisted of five rows of 2 m in length and 0.2 m distance between rows. The sowing rate was 20 g m^2^ and sowing dates were: 07 September and 23 May, in 2011 and 2012, respectively at Cauquenes; 31 and 7 August, in 2011 and 2012, respectively at Santa Rosa. Because the sowing date in 2011 at Cauquenes was much later than in 2012, the water stress was more severe in the first year. Plots were fertilized with 260 kg ha^1^ of ammonium phosphate (46% P_2_O_5_ and 18% N), 90 kg ha^−1^ of potassium chloride (60% K_2_O), 200 kg ha^−1^ of sul-po-mag (22% K_2_O, 18% MgO, and 22% S), 10 kg ha^−1^ of boronatrocalcite (11% B), and 3 kg ha^−1^ of zinc sulfate (35% Zn). Fertilizers were incorporated with a cultivator before sowing. During tillering an extra 153 kg ha^−1^ of N was applied. Weeds were controlled with the application of Flufenacet + Flurtamone + Diflufenican (96 g a.i.) as pre-emergence controls and a further application of MCPA (525 g a.i.) + Metsulfuron-metil (5 g a.i.) as post-emergents. Cultivars were disease resistance and no fungicide was used.

**Figure 1:**
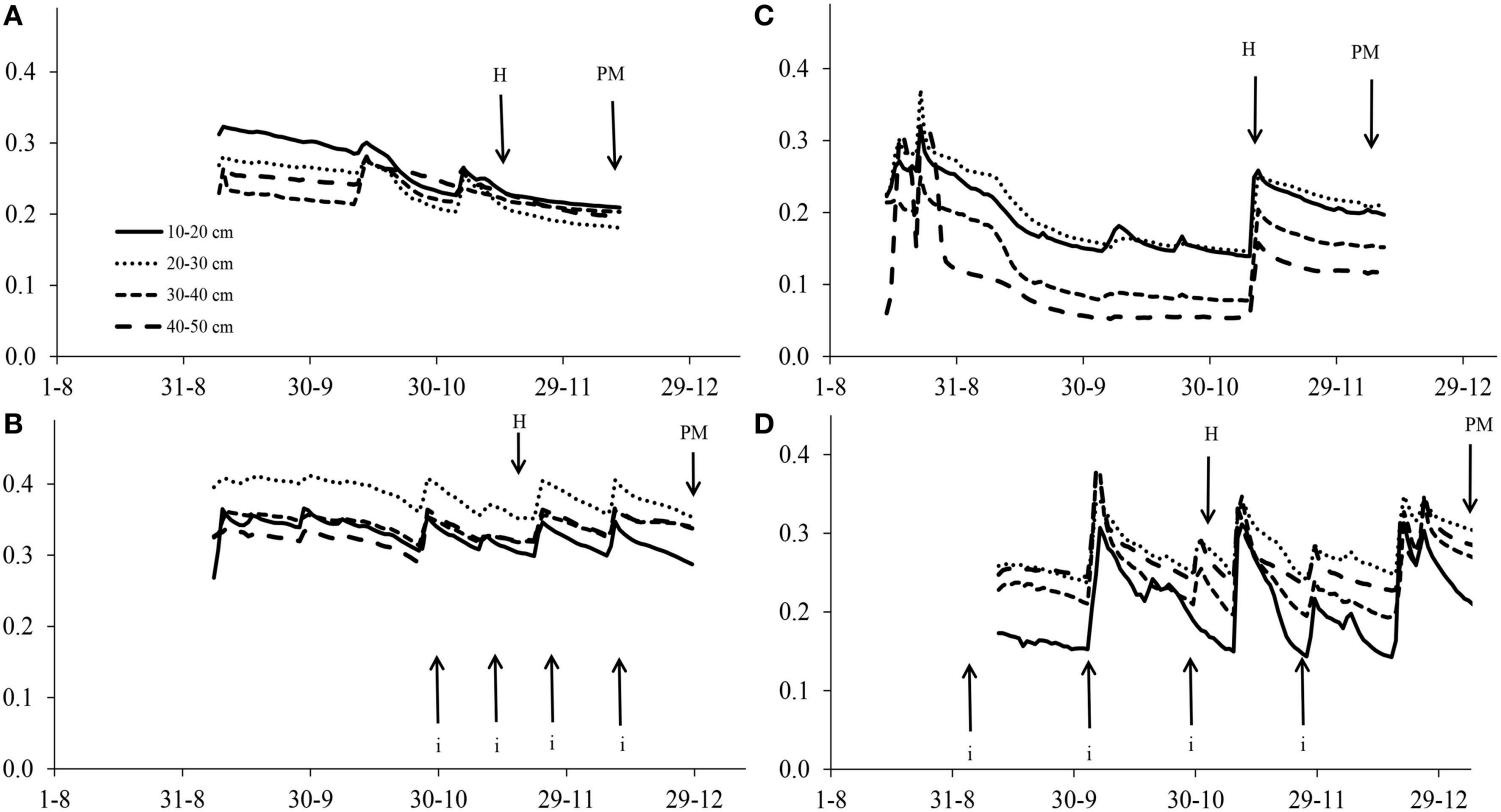
**Volumetric water content in the soil at severe water stress in Cauquenes (A,C) and at full irrigation in Santa Rosa (B,D) in 2011 (A,B) and 2012 (C,D)**. Each of the soil depth values are means from two sensors (replicates). Abbreviations H and PM refer to the dates of heading and physiological maturity, respectively. Dates of irrigation (i) at Santa Rosa are marked with arrows.

Furrow irrigation was used in Santa Rosa: one irrigation at the end of tillering (Zadocks Stage 21; Zadoks et al., 1974) in the MWS trial and four irrigations at the end of tillering, the flag leaf stage (Z37), heading (Z50), and middle grain filling (Z70) in the FI trial respectively. Soil moisture at 10–20, 20–30, 30–40, and 40–50 cm depth was determined by using 10HS sensors (Decagon Devices, USA) connected to an EM-50 data logger (Decagon Devices, USA). The 10HS sensor determines volumetric water content by measuring the dielectric constant of the soil using capacitance/frequency domain technology. Two sets of sensors were set up in each environment and mean values of two sensors per depth are presented in Figure 1.

### Agronomical traits

Days from emergence to heading (DH) were determined in Santa Rosa, through periodic (twice a week) observations, when approximately half of the spikes in the plot had already extruded. At maturity and for each plot the plant height (PH) of the different trials, up to the extreme of the spike (excluding awns), was measured, the number of spikes per m^2^ (SM2) were determined for a 1 m length of an inside row, and the number of kernels per spike (KS) and 1000 kernel weight (TKW) were determined in 25 spikes taken at random. Grain yield was assessed by harvesting the whole plot.

### Leaf chlorophyll content and water-soluble carbohydrates

Chlorophyll content (SPAD index) was determined at anthesis and then during grain filling about 2 weeks after anthesis (both measured on given calendar dates) in five flag leaves per plot using a SPAD 502 (Minolta Spectrum Technologies Inc., Plainfield, IL, USA) portable leaf chlorophyll meter. WSC concentration in stems (harvested at ground level and excluding leaf laminas and sheaths) was determined at anthesis and maturity, on five main stems per plot, using the anthrone reactive method (Yemm and Willis, 1954). The stem length was measured and then dried for 48 h at 60°C, weighed and ground. Next, a 100 mg subsample was used for WSC extraction, with 3 ml of extraction buffer containing 80% ethanol 10 mM Hepes-KOH (pH = 7.5), and incubated at 60°C overnight. Then, to separate the debris, the samples were centrifuged at 60 rpm for 30 min. The anthrone reagent was added to each supernatant and placed over a hotplate at 80°C for 20 min. Finally, the absorbance of the sample was measured at 620 nm in an EPOCH microplate UV-Vis Spectrophotometer (Biotek) using COSTAR 3636 96 well-plates (Corning) for the UV range. WSC content per whole stem and per unit land area were calculated as WSC concentration per unit stem weight (mg CHO g stem^−1^), and WSC content per unit of stem (mg CHO stem^−1^) and per unit grown area (g CHO m^−2^), respectively. In addition, the apparent WSC remobilization was calculated as the differences from anthesis to maturity in WSC content on a stem and land area basis.

### Stable carbon isotope analysis

The stable carbon (^13^C/^12^C) isotope ratio was measured in mature kernels using an elemental analyser (ANCA-SL, PDZ Europa, UK) coupled with an isotope ratio mass spectrometer, at the Laboratory of Applied Physical Chemistry at Ghent University (Belgium). The ^13^C/^12^C ratios were expressed in δ notation (Coplen, 2008) determined by: δ^13^C = (^13^C/^12^C)_sample_/(^13^C/^12^C)_standard_ −1 (Farquhar et al., 1989), where sample refers to plant material and standard to the laboratory standards that have been calibrated against international standards from Iso-Analytical (Crewe, Cheshire, UK). The precision of δ^13^C analyses was 0.3*%¸* (SD, *n* = 10). Further, the carbon isotope discrimination (Δ^13^C) of kernels was calculated as: Δ^13^C (*%¸*) = (δ^13^C_a_ − δ^13^C_p_)/[1+ (δ^13^C_p_)/1000], where a and p refer to air and the plant, respectively (Farquhar et al., 1989). δ^13^C_a_ from the air was taken as −8.0*‰*.

### Yield tolerance index

The yield tolerance index (YTI), which combines the relative performance of a genotype under drought with its potential yield under irrigated conditions (Ober et al., 2004), was calculated as:

(1) $$\begin{array}{r} YTI=\left(\frac{Y_{D}}{{\bar{Y}}_{D}}\right)\left(\frac{Y_{I}}{{\bar{Y}}_{I}}\right)\left(\frac{{\bar{Y}}_{D}}{{\bar{Y}}_{I}}\right)=\left(\frac{Y_{D}Y_{I}}{{\bar{Y}}_{I}^{2}}\right) \end{array}$$

where *Y*_*D*_ and *Y*_*I*_ are the genotype mean yield under drought (Cauquenes) and irrigation conditions (Santa Rosa, fully irrigation), respectively, and $\bar{Y}$_*D*_ and $\bar{Y}$_*I*_ are the mean yield of all genotypes growing under drought and irrigated conditions, respectively.

### Statistical analysis

In 2011, 10 genotypes were discarded from analysis due to low emergence. In addition six genotypes from Uruguay were discarded from the analysis for having late heading time (more than 100 days) an plant height >120 cm. ANOVAs for physiological and yield-related traits were performed for the whole set of genotypes using PROC MIXED of the SAS Institute Inc. Genotypes and environment (Cauquenes WS and Santa Rosa FI) were considered fixed effects, whereas blocks and incomplete blocks within each replication (in an α-lattice design) were considered random effects. Data from Santa Rosa MWS where not considered in the ANOVAs because there was no replication and only one year (2011) of observations. Correlation analysis was performed between agronomic and physiological traits, and also stepwise regressions between grain yield and related agronomical and physiological traits. Principal component analysis (PCA) was carried out for the 378 genotypes using the mean values for physiological and agronomical traits evaluated under severe water stress in Cauquenes and full irrigation in Santa Rosa, in two growing seasons, using IBM SPSS Statistics 19.

## Results

### Agronomical and physiological traits

For SM2, KS and TKW the genotype x environment (GxE) interaction was highly significant (*P* < 0.001) in both growing seasons, whereas for GY, PH, and KM2 was only in one growing season (Table 1). Among the physiological traits, the SPAD index exhibited a significant (*P* < 0.001) GxE interaction in both growing seasons, but stem weight and WSC concentration and content, and Δ^13^C of kernels was only in 2012 (Table 1).

**Table 1:** *****F***-values of ANOVA for agronomic and physiological traits, for 378 genotypes of wheat grown under severe water stress (Cauquenes WS) and full irrigation (Santa Rosa FI) in two growing seasons**.

**Agronomic traits**	**Year**	**Genotype (G)**	**Environment (E)**	**G × E**	**Physiological traits**	**Year**	**Genotype (G)**	**Environment (E)**	**G × E**
GY	2011^a^	1.2*	5001.9^***^	0.6	SPAD^a^	2011^a^	4.1^***^	211^***^	2.6^***^
	2012	2.5^***^	24217^***^	1.8^***^		2012	4.6^***^	4633^***^	2.0^***^
DH	2011	–	–	–	SPADgf	2011	–	–	–
	2012	–	–	–		2012	2.4^***^	1952^***^	1.8^***^
PH	2011	4.0^***^	12052^***^	1.4^**^	SW^a^	2011	0.7	76^***^	0.4
	2012	3.3^***^	543^***^	1.1		2012	5.3^***^	2398^***^	1.3^**^
SM2	2011	4.2^***^	1310^***^	1.4^**^	SWm	2011	0.5	128^***^	0.4
	2012	5.5^***^	7978^***^	2.1^***^		2012	6.2^***^	0.9	1.3^**^
KS	2011	6.6^***^	2383^***^	1.8^***^	WSC^a^	2011	1.3^*^	133^***^	1.0
	2012	3.8^***^	613^***^	1.6^***^		2012	1.2^*^	1249^***^	1.2^*^
TKW	2011	15.1^***^	6725^***^	1.4^**^	WSCm	2011	6.7^***^	2963^***^	6.6^***^
	2012	14.1^***^	906^***^	1.3^**^		2012	1.5^***^	13^***^	1.1
KM2	2011	2.2^***^	1860^***^	0.9	WSCC^a^	2011	0.7	3.8^*^	0.5
	2012	2.6^***^	6722^***^	1.7^***^		2012	2.4^***^	2305^***^	1.3^**^
					WSCCm	2011	1.3^*^	78^***^	1.1
						2012	1.8^***^	7.7^**^	1.2^**^
					Δ^13^C	2011	1.1	7512^***^	0.7
						2012	4.3^***^	45500^***^	1.6^***^

*GY, grain yield; DH, day to heading; PH, plant height; SM2, spikes m^−2^; KS, kernel per spike; TKW, thousand kernel weight; KM2, kernels m^−2^; SPADa, SPAD anthesis; SPADgf, SPAD grain filling; SWa, stem weight at anthesis; SWm, stem weight at maturity; WSCa, WSC at anthesis; WSCm, WSC at maturity; WSCCa, WSC content anthesis; WSCCm, WSC content maturity; Δ^13^C, kernel Δ^13^C*.

a *In 2011, 10 genotypes were discarded from the analysis due to low spike numbers*.

* P < 0.05;

** P < 0.001;

*** *P < 0.0001*.

**Figure 2:**
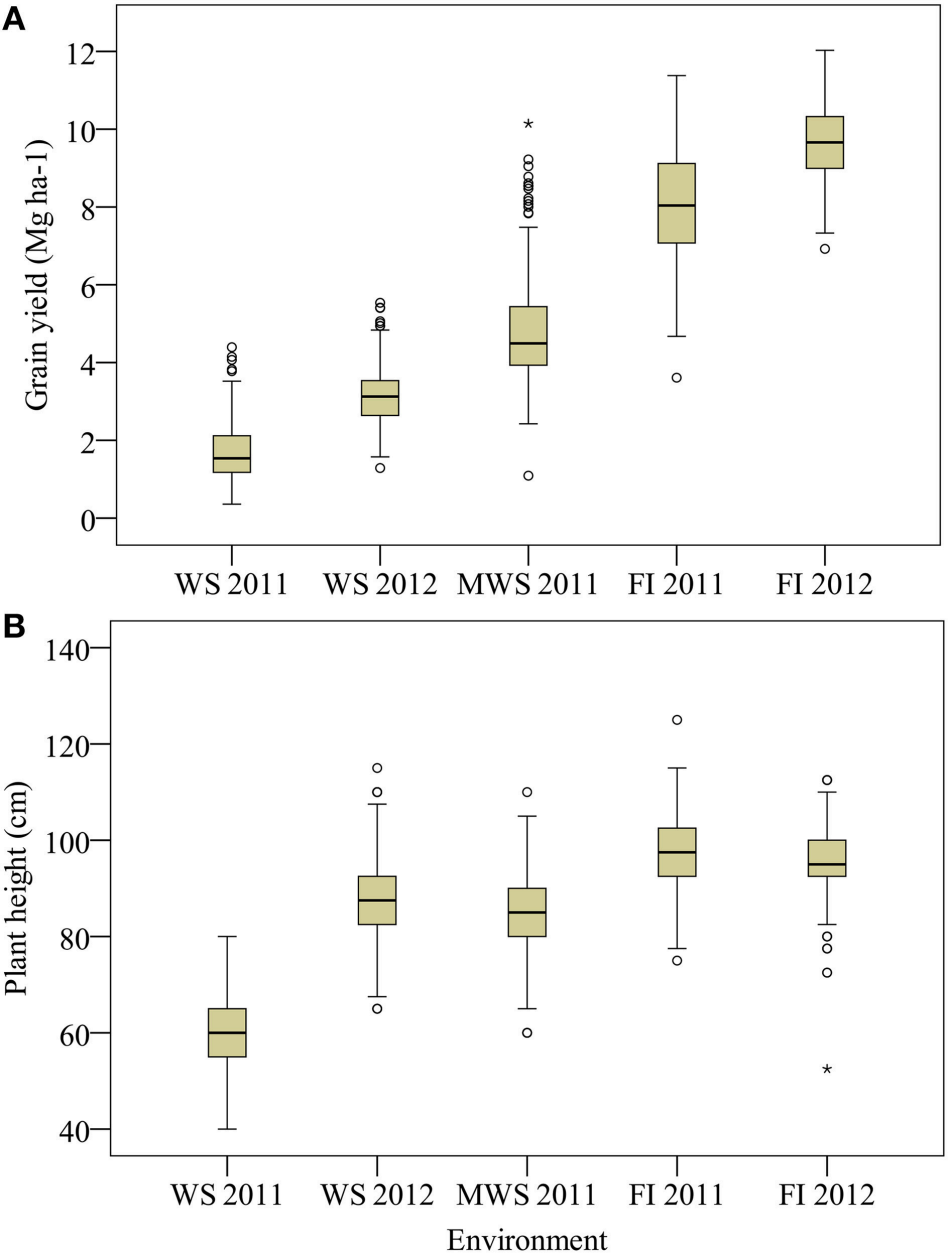
**Grain yield (A) and plant height (B) for 378 genotypes of wheat grown under water stress (Cauquenes WS), mild water stress (Santa Rosa MWS) and full irrigation (Santa Rosa FI) in two growing seasons (2011 and 2012), except at MWS**. Box and whisker show population minimum, 25th percentile/median/75th percentile and maximum. The open symbols indicate outlier data.

Under FI in Santa Rosa, the average GY of the three sets of wheat genotypes (378 in total) was 8–10 Mg ha^−1^ but some genotypes produced up to 12 Mg ha^−1^ (Figure 2A). Under MWS in Santa Rosa the average GY was 4.8 Mg ha^−1^. Under WS GY was significantly (*P* < 0.0001) reduced in Cauquenes, by 79 and 68% in 2011 and 2012, respectively, compared to Santa Rosa under FI (Figure 2A). Also, plant height was reduced under WS by 40 and 9% in 2011 and 2012, respectively (Figure 2B).

**Figure 3:**
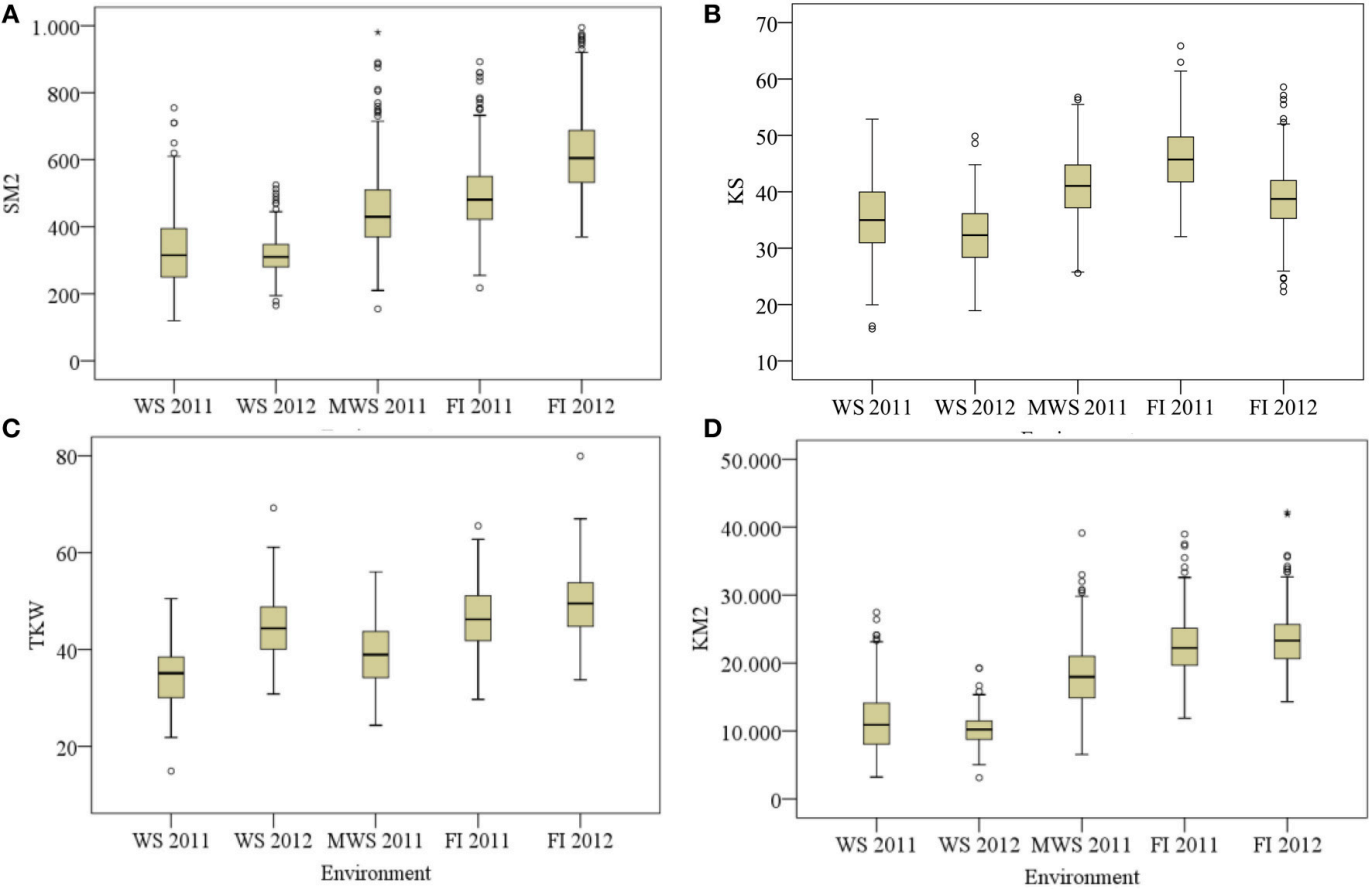
**Spikes per m^**2**^ (SM2; A), kernel per spike (KS; B), thousand kernel weight [TKW (g); C] and kernels m^**−2**^ (KM2; D) for 378 genotypes of wheat grown under water stress (Cauquenes WS), mild water stress (Santa Rosa MWS) and full irrigation (Santa Rosa FI) in two growing seasons (2011 and 2012), except at MWS**. Box and whisker show population minimum, 25th percentile/median/75th percentile and maximum. The open symbols indicate outlier data.

The reduction in SM2, KS and TKW under WS compared with FI was in general more pronounced in the first growing season; on average (of the two growing seasons) these traits were reduced by 25, 41, 21, and 18%, respectively, whereas KM2 was reduced by 53% (Figure 3).

The relationships for GY under FI and WS showed no significant correlation in both years (*P* > 0.05). The yield tolerance index (YTI) of the 378 genotypes based on GY under WS and FI presented a wide range of values in both years, from 0.05 (very susceptible) to 0.65 (very tolerant genotypes). The frequency distribution of YTI had a left-skewed deviation in 2011 (mean YTI = 0.21) compared to 2012 (mean YTI = 0.32).

Days to heading, determined under FI, differed by about 20 days between the earliest and latest genotypes (Table 2). A wide range of SPAD index values among genotypes was observed in environments (WS, MWS, and FI) and growing seasons (Table 2). A significant reduction (*P* < 0.001) in the SPAD index at anthesis and during grain filling was observed under WS in 2012.

**Table 2:** **Means ± standard deviation and ranges (minimum–maximum) for chlorophyll content in SPAD units, stem weight, water-soluble carbohydrate (WSC) concentration and content per stem at anthesis and maturity, and carbon isotope discrimination (Δ^**13**^C) in kernels, for 378 genotypes of wheat grown under severe water stress (Cauquenes WS), mild water stress (Santa Rosa MWS) and full irrigation (Santa Rosa FI) in two growing seasons, except at MWS**.

		**Cauquenes (WS)**		**Santa Rosa (MWS)**		**Santa Rosa (FI)**	
**Trait**	**Year**	**Mean**	**Range**	**Mean**	**Range**	**Mean**	**Range**
Days to heading	2011	n.e.	n.e.	78 ± 3	71–86	79 ± 3	73–89
	2012	n.e.	n.e.	n.e.	n.e.	85 ± 4	78–100
SPAD anthesis	2011	47 ± 5	32–60	47 ± 3	34–56	45 ± 3	36–52
	2012	42 ± 3	32–50	n.e.	n.e.	49 ± 3	41–58
SPAD grain filling	2011	n.e.	n.e.	31 ± 12	4–52	43 ± 3	30–51
	2012	34 ± 8	4–47	n.e.	n.e.	48 ± 3	35–55
Stem weight anthesis (g)	2011	1.24 ± 0.31	0.50–2.44	1.68 ± 0.40	0.86–2.88	1.67 ± 0.36	0.91–2.84
	2012	1.81 ± 0.36	0.91–3.04	n.e.	n.e.	1.19 ± 0.27	0.54–1.95
Stem weight maturity (g)	2011	0.70 ± 0.19	0.26–1.48	0.98 ± 0.26	0.41–3.03	1.14 ± 0.25	0.57–2.06
	2012	1.06 ± 0.24	0.59–1.79	n.e.	n.e.	1.06 ± 0.23	0.54–2.13
WSC concentration anthesis (mg g^−1^)	2011	178.4 ± 56.0	63.9–548.3	152.6 ± 40.7	74.0–431.1	139.9 ± 41.7	29.1–349.7
	2012	226.7 ± 36.2	110.5–444.3	n.e.	n.e.	142.3 ± 38.3	64.3–381.7
WSC concentration maturity (mg g^−1^)	2011	43.4 ± 23.2	15.8–171.3	36.4 ± 26.7	3.7–224.2	17.7 ± 5.4	7.0–32.7
	2012	48.5 ± 18.1	20.7–146.6	n.e.	n.e.	41.7 ± 17.8	14.7–153.5
Total WSC content anthesis (mg stem^−1^)	2011	221.5 ± 91.5	49.8–770.6	256.8 ± 92.3	80.8–758.7	234.9 ± 95.5	58.6–636.4
	2012	413.2 ± 110.5	158.0–988.9	n.e.	n.e.	174.2 ± 73.1	37.8–583.5
Total WSC content maturity (mg stem^−1^)	2011	30.6 ± 19.6	7.3–157.7	36.9 ± 33.0	3.3–291.5	20.2 ± 8.4	3.2–53.2
	2012	53.1 ± 27.7	17.1–169.9	n.e.	n.e.	43.9 ± 24.7	6.5–195.1
Kernel Δ^13^C (^0^/00)	2011	14.2 ± 0.6	11.9–16.3	16.5 ± 0.9	14.6–19.6	18.1 ± 0.6	16.2–19.6
	2012	14.9 ± 0.4	13.4–16.1	n.e.	n.e.	18.7 ± 0.4	17.4–20.1

Stem weight and stem WSC concentration and content were much higher at anthesis compared to maturity. Their average reductions over two growing seasons were about 43, 77, and 87%, respectively, under WS at Cauquenes, and 23, 79, and 84%, respectively, in Santa Rosa under FI (Table 2). The apparent WSC remobilization was on average 279, 220, and 170 mg per stem under WS, MWS, and FI, respectively (data not shown).

The WSC concentration and content per stem at anthesis and maturity presented large genotypic variabilities in all the environments (Table 2). The stem WSC per unit area (g m^−2^) at anthesis was highly correlated to the WSC concentration (*r* = 0.66 and 0.84, *P* < 0.001, for WS for FI, respectively, in 2012) and the stem biomass (g m^−2^; *r* = 0.81 and 0.66, *P* < 0.001, for a WS for FI, respectively, in 2012).

The Δ^13^C also exhibited genotypic variability under WS, MWS, and FI (Table 2), but lower values were found under WS compared to FI.

### Relationships between yield, agronomical, and physiological traits

**Figure 4:**
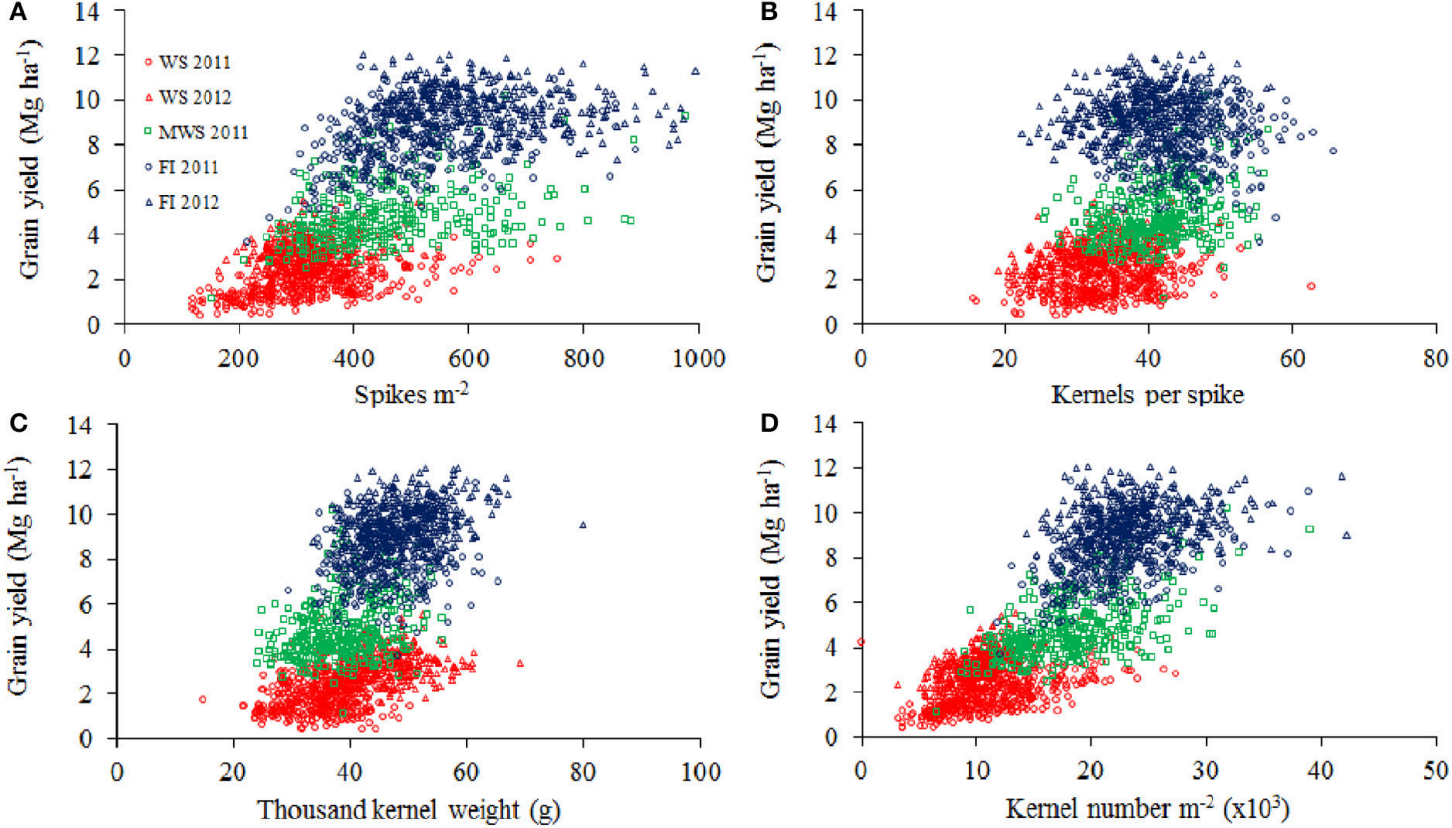
**Table:** 

GY was positively correlated with SM2 and KM2, but negatively correlated with TKW, in both water regimes and growing seasons (Figure 4). GY was also positively correlated (*r* = 0.3–0.52, *P* < 0.001) with plant height in all the environments.

	**Grain yield**				
	**WS 2011**	**WS 2012**	**MWS 2011**	**FI 2011**	**FI 2012**
Spikes m^−2^	**0.60**	**0.10**	**0.35**	**0.30**	−**0.14**
Kernels spike^−1^	**0.47**	**0.51**	**0.27**	**0.19**	**0.35**
Thousand kernel weight	0.01	**0.32**	**0.29**	**0.13**	**0.28**
Kernels m^−2^ (x10^3^)	**0.75**	**0.46**	**0.53**	**0.50**	**0.16**

Coefficients of significant correlations (at *P* < 0.05) are in bold characters. **Relationships between grain yield and spikes per m^**−2**^ (A), kernel per spike (B), thousand kernel weight (C), and kernel number per m^**−2**^ (D) of 378 genotypes of wheat grown under water stress (WS) in Cauquenes and mild water stress (MWS) and full irrigation (FI) in Santa Rosa, in 2011 and 2012**. Pearson correlation values are shown in the table above.

**Figure 5:**
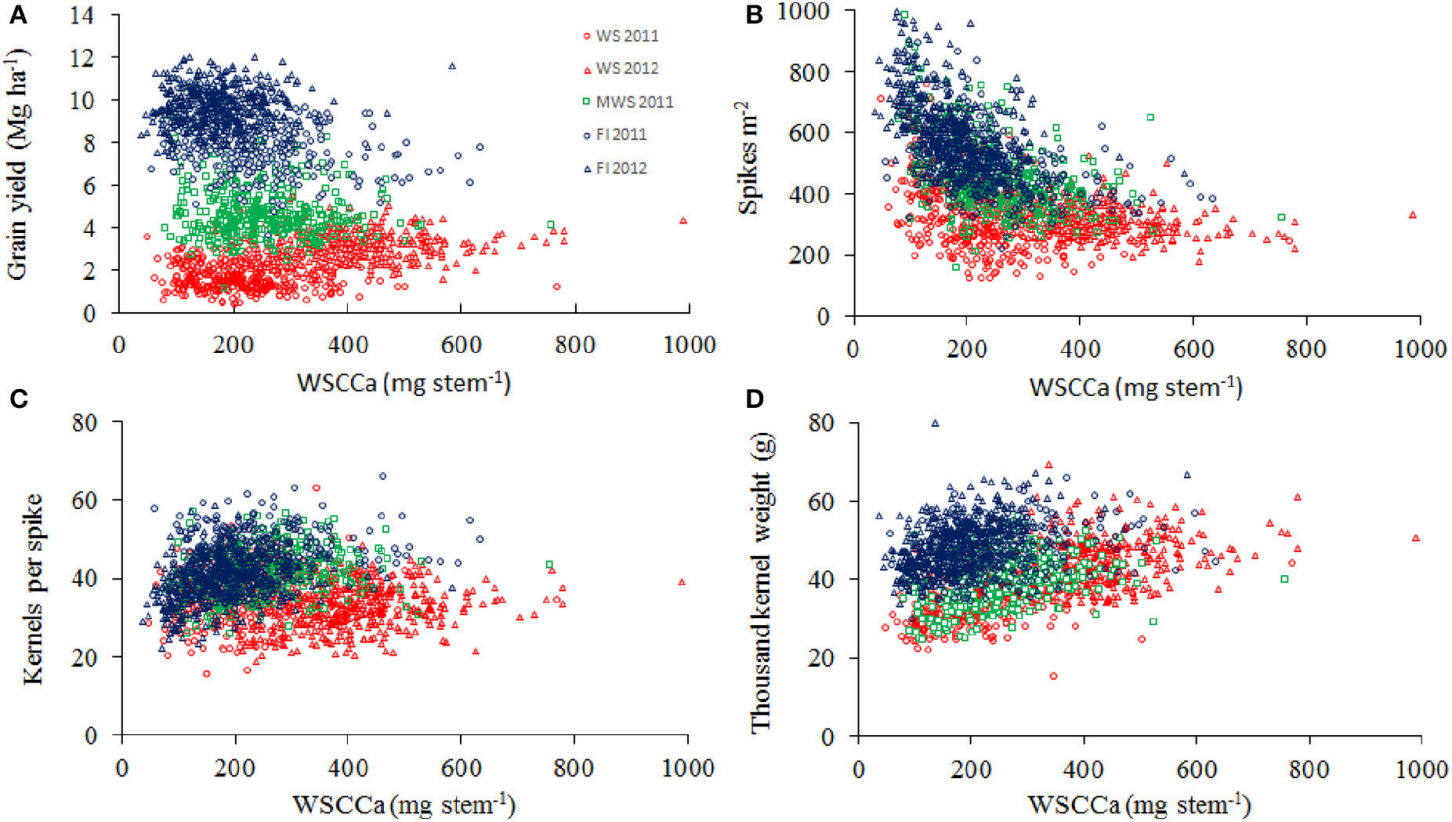
**Relationships between stem water-soluble carbohydrate content at anthesis (WSCCa) and grain yield (A), spikes per m^**−2**^ (B), kernel per spike (C), and thousand kernel weight (D) in 378 genotypes of wheat grown under water stress (WS) in Cauquenes and mild water stress (MWS) and full irrigation (FI) in Santa Rosa, in 2011 and 2012**. Pearson correlation values are in Table 3.

Days to heading (determined at FI) was not correlated with GY, but it was positively correlated with SM2 and negatively correlated with TKW, except under FI in 2012 (Table 3). The SPAD index was positive and significantly correlated with GY (except under FI in 2011) and the agronomical components KS and TKW (Table 3). The WSC content at anthesis (WSCCa) was negatively correlated with SM2, but positively correlated with KS and TKW under WS and FI conditions (Figure 5). As a consequence, GY exhibited a low positive correlation with WSCCa under WS in 2012, and non or negative correlation under FI (Table 3).

**Table 3:** **Correlations between physiological traits and grain yield and their agronomical components of 378 genotypes of wheat grown under water stress (WS) in Cauquenes and full irrigation (FI) in Santa Rosa, in 2011 and 2012**.

	**Grain yield**				**Spikes m**^−2^				**Kernels spike**^−1^				**Thousand kernel weight**			
	**WS 2011**	**WS 2012**	**FI 2011**	**FI 2012**	**WS 2011**	**WS 2012**	**FI 2011**	**FI2012**	**WS 2011**	**WS 2012**	**FI 2011**	**FI2012**	**WS 2011**	**WS 2012**	**FI 2011**	**FI 2012**
DH	−0.03	0.08	−0.02	**0.17**	**0.18**	**0.16**	**0.35**	**0.32**	**0.12**	0.02	0.02	0.08	−**0.54**	−**0.35**	−**0.43**	0.08
SPADa	**0.39**	**0.32**	−0.04	**0.22**	**0.15**	−**0.27**	−**0.43**	−**0.38**	**0.41**	**0.21**	**0.25**	**0.23**	**0.10**	**0.36**	**0.44**	**0.36**
SPADgf	n.e.	**0.19**	0.09	**0.24**	n.e.	−**0.21**	−**0.33**	−**0.30**	n.e.	0.05	**0.30**	**0.21**	n.e.	**0.33**	**0.41**	**0.29**
SWD	0.03	0.08	−**0.18**	0.00	−**0.26**	−**0.21**	−**0.39**	−**0.26**	**0.21**	**0.29**	**0.17**	**0.17**	**0.28**	**0.10**	**0.34**	**0.19**
WSCa	−**0.26**	−0.09	−**0.37**	−**0.14**	−**0.32**	0.05	−**0.10**	−**0.28**	−0.02	−0.02	−0.07	0.09	0.05	−**0.11**	−0.09	**0.25**
WSCm	−**0.28**	−0.02	−**0.12**	0.00	−**0.22**	−**0.12**	−**0.14**	0.06	−**0.33**	−0.09	−0.01	−**0.14**	**0.18**	**0.15**	**0.12**	0.02
WSCCa	−0.07	**0.15**	−**0.33**	0.05	−**0.41**	−**0.30**	−**0.45**	−**0.53**	**0.22**	**0.28**	**0.19**	**0.27**	**0.38**	**0.31**	**0.30**	**0.50**
WSCCm	−**0.13**	**0.14**	−0.06	0.11	−**0.24**	−**0.27**	−**0.43**	−**0.17**	−**0.11**	0.06	**0.26**	0.03	**0.38**	**0.39**	**0.43**	**0.22**
WSCCD	−0.03	**0.12**	−**0.33**	0.01	−**0.38**	−**0.25**	−**0.42**	−**0.47**	**0.27**	**0.28**	**0.16**	**0.26**	**0.30**	**0.22**	**0.28**	**0.43**
Δ^13^C	−**0.12**	**0.53**	**0.51**	**0.42**	−0.06	0.09	0.05	−0.07	−0.01	**0.26**	**0.23**	**0.21**	**0.16**	**0.11**	**0.21**	**0.13**

*Coefficients of significant correlations (at P < 0.05) are in bold characters. HD, day to heading; SPADa, SPAD at anthesis; SPADgf, SPAD at grain filling; SWD, stem weight difference (SWa-SWm); WSCa, WSC at anthesis; WSCm, WSC at maturity; WSCCa, WSC content at anthesis; WSCCm, WSC content at maturity; Δ^13^C, kernel Δ^13^C. n.e.: not evaluated*.

**Figure 6:**
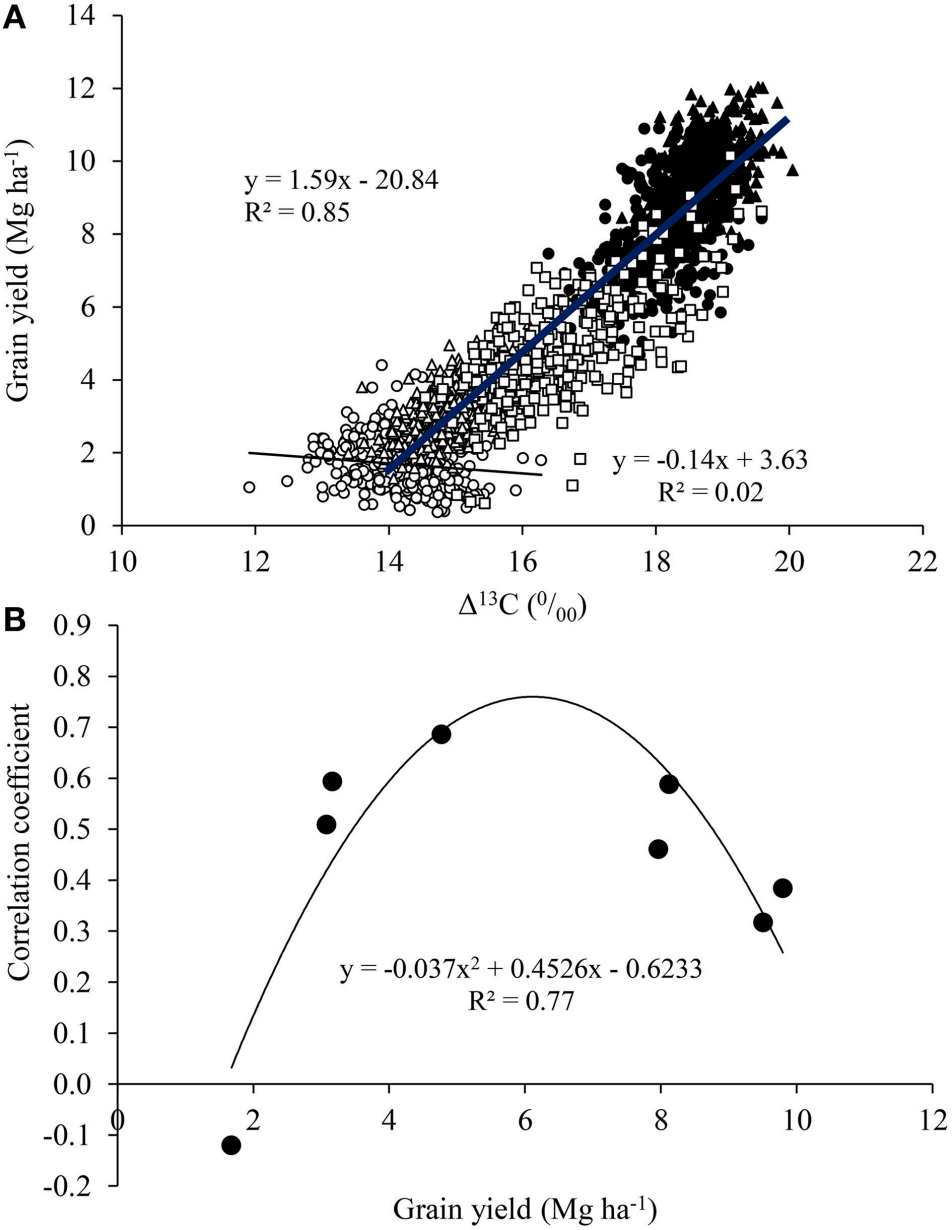
**(A)** Relationships between grain yield (GY) and carbon discrimination in grains (Δ^13^C) for the set of 384 spring wheat genotypes grown under water stress (○, Δ), mild water stress (□) and full irrigation (•, ▴) conditions in 2011 and 2012. The negative regression is for water stress in 2011 and the positive one is for the rest of the environments. **(B)** The relationship between GY and correlation coefficients between GY and Δ^13^C for each replicate (block) and environment (WS, MWS and FI in 2011, and WS and FI in 2012).

The relationship between Δ^13^C and GY was slightly negative under WS in 2011, but positive and highly significant in 2012, and also positive under MWS and FI in 2011 and 2012 (Table 3; Figure 6A). Indeed, Pearson correlation values of the relationship between Δ^13^C vs. GY depended on the environment, increasing from low to medium yields and further declining at higher GY (Figure 6B). The correlation between Δ^13^C and STI under SWS was not significant in 2011 but was positive and significant in 2012 (*r* = 0.51; *P* < 0.01).

**Figure 7:**
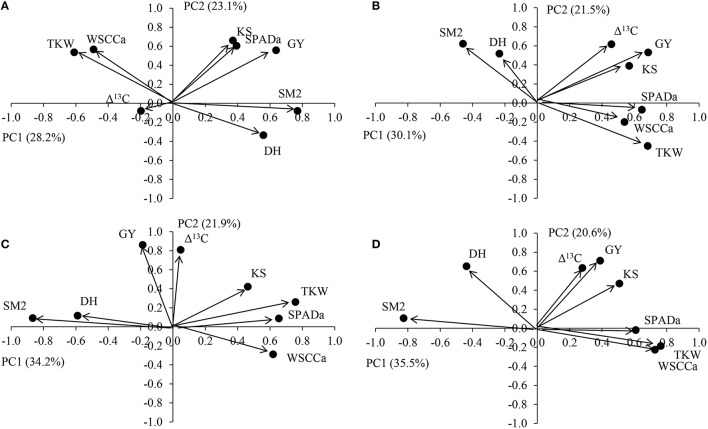
**PCA analysis of physiological and agronomical traits measured in 378 genotypes of wheat grown under severe water stress in Cauquenes (A,B) and full irrigation in Santa Rosa (C,D), in 2011 (A,C) and 2012 (B,D)**. Traits included in the PCA are day to heading (DH), grain yield (GY), kernel per spike (KS), thousand kernel weight (TKW), spikes per kernels m^−2^ (KM2), chlorophyll content at anthesis (SPAD), water soluble carbohydrates content at anthesis (WSCCa), and carbon isotope discrimination (Δ^13^C) in kernels.

PCA analysis indicated that the two first principal components (PC) explained >50% of the observed variability, under WS and FI conditions (Figure 7). KS was the agronomical component more close related with GY under WS and FI (except in 2011). Among the physiological traits, Δ^13^C presented the strongest association with GY, except under the severe WS in 2011 (Figure 7). The SPAD index at anthesis was close associated with GY under WS in 2011, but with TKW under WS in 2012 and FI. WSCCa was also close related to TKW in all the environments, and days to heading was associated SM2.

The stepwise regression analysis between GY and related agronomical (SM2, TKW, and KS) and physiological (SPADa, WSCCa, and Δ^13^C) traits indicated that under water stress conditions, the contribution of the agronomical trails was greater than the physiological ones, but under full irrigation conditions WSCCa and Δ^13^C contributed similarly to the agronomical traits to GY (Table 4).

**Table 4:** **Stepwise regression analysis between grain yield (GY) and related agronomical (TKW, SM2, and KS) and physiological (SPADa, WSCCa, and Δ^**13**^C) traits of 378 genotypes of wheat grown under water stress (WS) in Cauquenes and full irrigation (FI) in Santa Rosa, in 2011 and 2012**.

	**WS 2011–2012**				**FI 2011–2012**			
**Trait**	**Coefficient B**	**SE**	**Contribution**		**Coefficient B**	**SE**	**Contribution**	
			**Share**	**Unique**			**Share**	**Unique**
Constant	−7.218	0.618			−19.270	1.138		
TKW	0.068	0.004	30.9	13.9	0.089	0.007	16.8	8.1
SM2	0.006	0.000	39.2	19.9	0.005	0.000	16.4	7.9
KS	0.049	0.004	18.6	7.1	0.072	0.007	12.6	5.8
WSCCa	0.002	0.000	7.5	2.5	−0.004	0.000	16.8	8.1
Δ^13^C	0.281	0.039	6.6	2.2	0.878	0.068	18.7	9.2
SPADa	−0.026	0.005	3.4	1.1	0.051	0.011	2.8	1.2
*R*^2^	0.69				0.60			
*N*	722				734			
Mean square	84.21				183.14			
*P*	< 0.0001				*P* < 0.0001			

## Discussion

The set of 378 wheat genotypes tested in this work exhibited a high phenotypic variability for physiological and agronomic traits. The water stress in Cauquenes was very severe as reflected in the low average GY (1.7 Mg ha^−1^ in 2011). However, some genotypes were able to produce more than 4 Mg ha^−1^ under such WS conditions and showed high values of YTI (>0.50). Actually, YTI was highly correlated (*r* > 0.92; *P* < 0.0001 in both years) with GY under WS in Cauquenes. Under the full irrigation conditions of Santa Rosa some genotypes achieved extremely high yields (12 Mg ha^−1^), for a Mediterranean environment. Large genotypic variability in GY and its agronomical components has also been found in 127 recombinant inbred lines (Dharwar Dry × Sitta) of wheat growing under severe water stress in Obregon, Mexico (Kirigwi et al., 2007), and in 105 lines of the double-haploid population (Weebil × Bacanora) in four contrasting high-yielding environments (García et al., 2013).

The strong reduction in GY under WS was mainly a consequence of the decline in SM2 (41%), followed by KS (21%), and as a consequence the number of kernels per m^2^ was reduced (53%; Table 2). Thus, kernels per m^2^ is the agronomical component most affected by drought, as previously reported by other authors (Estrada-Campuzano et al., 2012). In addition the TKW also decreased, but to a lesser extent (18%). As a consequence GY was positively correlated with the number of kernels m^−2^ (Figure 4; *r* = 0.81, *P* < 0.0001 for all the environments), but the correlation coefficients for each environment were not as high as has been reported by several authors (see Sinclair and Jamieson, 2006). In fact, a trade-off among the agronomical components was observed where SM2 was negatively correlated with KS under FI (*r* = −0.50 and −0.58 in 2011 and 2012, respectively) and TKW in WS (*r* = −0.36 and −0.49 in 2011 and 2012, respectively) and FI (*r* = −0.60 and −0.58 in 2011 and 2012, respectively) conditions. The PCA indicated that KS was better associated with GY in both WS and FI conditions (Figure 7). Other studies have also shown that KS but not TKW was associated with GY under water stress conditions (Denčić et al., 2000) and also a high-yielding environment (García et al., 2013).

### Chlorophyll content

Chlorophyll content at anthesis was positively correlated with GY and the agronomical components KS and TKW, particularly under WS (Table 3). Drought increases senescence by accelerating chlorophyll degradation leading to a decrease in leaf area and photosynthesis. There is evidence that stay-green phenotypes with delayed leaf senescence can improve their performance under drought conditions (Rivero et al., 2007; Lopes and Reynolds, 2012). In wheat and sorghum, genotypic variability has been detected in chlorophyll content as well as in the rate of leaf senescence (measured with a portable leaf chlorophyll meter) during grain-filling (Harris et al., 2007; Lopes and Reynolds, 2012). In durum wheat (*Triticum turgidum* ssp. durum) stay-green mutants growing under glasshouse conditions remained green for longer and had higher rates of leaf photosynthesis and seed weight (Spano et al., 2003). These mutants with the stay-green characteristic also had higher levels of expression of the Rubisco small subunit of (RBCS) and chlorophyll a/b binding protein (Rampino et al., 2006). Bread wheat genotypes with functional stay-green characteristics have also shown higher GY and total biomass in field conditions (Chen et al., 2010). Another study on Canadian spring wheat revealed that GY was positively correlated with green flag leaf duration and total flag leaf photosynthesis (Wang et al., 2008). Studies on spring wheat in the USA found a positive correlation between the stay-green trait and GY and grain weight in both water-limited and well-watered conditions (Blake et al., 2007). Therefore, a delay in leaf senescence would increase the amount of fixed carbon available for grain filling.

### Stem water-soluble carbohydrate

Large genotypic variability in stem WSC concentration and content was found at anthesis and maturity, in both environments (Table 2; Figure 5). Other studies conducted in spring and winter wheat lines have also found large variability in WSC concentration and WSC content on an area basis in stems around the time of anthesis (Ruuska et al., 2006; Foulkes et al., 2007; Yang et al., 2007). WSCs are accumulated in stems prior to anthesis and are then remobilized to the grain during the grain-filling period (Blum, 1998; Bingham et al., 2007). Indeed under water limiting conditions, where canopy photosynthesis is inhibited, the contribution of stem carbohydrate to grain growth could be very significant (Ehdaie et al., 2006a,b; Reynolds et al., 2006). In our study, more carbohydrate was accumulated at anthesis under WS than under FI, and the decline in stem WSC from anthesis to maturity was greater under WS, particularly in 2012 (360 vs. 130 mg per stem under WS and FI, respectively). This suggests that there was a larger remobilization of reserves during grain filling under WS. However, there were no clear relationships between the stem WSCCa, or the apparent WSC remobilization and GY, varying the correlation values from not significant to negative on the different environments (Table 3; Figure 5). Zhang et al. (2015) found also no significant correlation between stem WSC and GY in 20 genetically diverse double haploids derived from the cross of cvs. Westonia × Kauz, growing under drought, and irrigated conditions in Western Australia. These results differ from those found by Foulkes et al. (2007) in winter wheat under non water-stressed conditions in England.

It seems that there is a trade-off between the stem WSCCa and some of the agronomical yield components. In fact, negative correlations exist with SM2 in all the environments, but the correlations were positive with KS and TKW (Table 3; Figure 5). The PCA analyses also showed a high association between WSCCa and TKW (Figure 7). This negative relationship between WSC and either number of stems or number spikes per m^2^ at maturity has also been reported for other wheat genotypes (Rebetzke et al., 2008a; Dreccer et al., 2009, 2013). Why genotypes with lower number of stems present higher stem WSC concentration and content? A possible explanation is that genotypes with lower number of stems per unit area have bigger stems; if fact, our results indicated a significant (*p* < 0.001) negative correlation (*r* = −0.29 and −0.36 under WS, and −0.58 and −0.56 under FI, in 2011 and 2012, respectively) between SM2 and stem weight at anthesis. Thus, genotypes with lower number of stems have probably more light transmission through the canopy and therefore higher rates of photosynthesis per stem, leading to higher stem weight and WSC content (more reserves), and greater numbers of grains per spike and kernel size. A significant and positive correlation between accumulated WSC at anthesis and kernel weight has been also observed in recombinant inbred lines (RILs) from the Seri/Babax population (Dreccer et al., 2009). Another hypothesis (complementary of the previous one) may be that those genotypes able to produce less tillers (because poorer adaptation to growing conditions—such as water stress-) are those which accumulate more carbohydrate since these photo-assimilates are not used for growth. Therefore, selecting for high stem WSC, either under near optimal agronomical conditions or under water stress, would probably lead to genotypes with lower tillering capacity and GY potential. The study conducted by Dreccer et al. (2013) in RILs of contrasting tillering and WSC concentration in the stem, and grown at different plant densities or on different sowing dates, indicates that genotypic rankings for stem WSC persisted when RILs were compared at similar stem density.

### Carbon isotope discrimination

The genotypic differences in carbon isotope discrimination found among the 384 genotypes (Table 3) agree with other studies conducted in Mediterranean conditions. For example, higher Δ^13^C (or lower carbon isotope composition, δ^13^C) in modern cultivars compared with old varieties has been found in bread (del Pozo et al., 2014) and durum wheats (Araus et al., 2013).

The relationship between Δ13C and GY was positive under MWS or FI but was negative under WS (Figure 5). Other studies in wheat (Araus et al., 2003, 2008) and barley (del Pozo et al., 2012) have also shown that Δ^13^C in kernels can be positively or negatively correlated with GY depending on soil water availability. Positive relationships between Δ^13^C (or negative with δ^13^C) and GY have been frequently reported for cereals under Mediterranean conditions (see Rebetzke et al., 2008b for bread wheat and Araus et al., 2003, 2013 for durum wheat), and this can be explained by the fact that genotypes maintaining a larger transpiration and thus water use during the crop cycle will be the most productive (Araus et al., 2003, 2008, 2013; Blum, 2005, 2009). In fact, negative relationships between kernel oxygen isotope composition (δ^18^O) or enrichment (Δ^18^O) and grain yield have been reported in bread wheat under fully irrigated conditions (Cabrera-Bosquet et al., 2011; del Pozo et al., 2014) as well as for durum wheat under Mediterranean conditions (Araus et al., 2013) and subtropical maize under well irrigated and moderate stress (Cabrera-Bosquet et al., 2009). Indeed, carbon isotope composition can be used as a selection criterion for high water use efficiency (Condon et al., 2004; Richards, 2006), but also can provide an indirect determination of the effective water used by the crop (Araus et al., 2002, 2008; Blum, 2009). The effect of phenology on Δ^13^C (earlier genotypes exhibiting higher Δ^13^C) may be discarded, since heading date was not correlated with Δ^13^C (*P* > 0.05) in none of the environments. Actually, the positive correlations between Δ^13^C and GY was also found when the relationship were studied within subset of 212 genotypes with similar heading duration (80–85 days); *r* = 0.50 for WS and 0.42 for WI in 2012.

## Conclusions

The identification of genotypic variability for agronomical and physiological traits under water stress conditions and full irrigation is of great interest for breeders because selected genotypes with favorable traits can be used as parents in future crosses. Among these, genotypes with higher numbers of fertile tillers would lead to higher numbers of kernels per m^2^ and GY under terminal water stress and non-stress conditions. Additionally, genotypes with delay in leaf senescence (a higher SPAD index) would lead to higher KS and TKW, particularly under water stress, and to a lesser extent at full irrigation. In the case of yield potential conditions, this is probably the consequence of greater amounts of fixed carbon available for grain filling, whereas under water stress stay-green it is an indicator of resilience to stress conditions. In addition, genotypes with higher carbon discrimination values are associated with higher GY under MWS and full irrigation, indicating that more water is used by the crop. In addition, selection for a higher WSC at anthesis may bring negative consequences in terms of yield potential and adaptation to MWS conditions. This study clearly illustrates the importance of defining the target environment for wheat breeding before determining the set of phenotyping traits for selection.

## Author contributions

AD and IM designed the experiments, selected the germplasm and participated on field evaluations. AY and GT were in charge of carbohydrate determinations. DC was in charge of the management of the experiments and evaluation of agronomic traits. LS and JA contributed to analysis of the data. AD was in charge of the writing up but all the authors contributed to the manuscript.

### Conflict of interest statement

The authors declare that the research was conducted in the absence of any commercial or financial relationships that could be construed as a potential conflict of interest.
