# Supplementary material for: Physiological Traits Associated with Wheat Yield Potential and Performance under Water-Stress in a Mediterranean Environment
Source: Front Plant Sci. 2016 Jul 7;7:987. doi: 10.3389/fpls.2016.00987 (PMC4936474; doi:10.3389/fpls.2016.00987)
Supplement: Supplementary file 1 [file Table1.DOCX]

**Supplementary information**

Table S1. List of the 384 spring wheat genotypes from INIA-Chile, CIMMYT and INIA-Uruguay used in this study and mean values (2011 and 2012) of yield tolerance index (YTI).

| FA | Name | Pedigree | Origen | YTI |
| --- | --- | --- | --- | --- |
| 1 | Pandora | TJB358.251/BUC//CIKO | Chile | 0.36 |
| 2 | Pantera | TJB358.251/BUC//CIKO | Chile | 0.49 |
| 3 | Kipa | QUP 1867_91/DOMO | Chile | 0.33 |
| 4 | Millan | VS73.600/MRL’S’/3/BOW’S’//YR/TRF’S’/4/CIKO | Chile | 0.24 |
| 5 | Libungo | LIBUN E.B./SONKA/4/BEZ/7C//LIKAY/3/NESMA,BT.149 | Chile | 0.30 |
| 9 | QUP2405_2006 | QUP 1865_96/CAR3911//QUP 1865_96 | Chile | 0.34 |
| 8 | QUP2409_2006 | FILIN/DOMO | Chile | 0.34 |
| 12 | QUP2418_2007 | ALTAR84/AE.SQUA (221)//SIREM/3/SRMA/TUI | Chile | 0.37 |
| 18 | QUP2419_2009 | HD2206/HORK//BUC/BUL/3/QUP 1619_95 | Chile | 0.32 |
| 47 | QUP2421_2009 | HD2206/HORK//BUC/BUL/3/QUP 1619_95 | Chile | 0.46 |
| 48 | QUP2422_2009 | TAMOI/QUP 1619_95 | Chile | 0.33 |
| 49 | QUP2423_2009 | PASTOR/DOMO | Chile | 0.55 |
| 50 | QUP2424_2009 | MILAN/PASTOR//DOMO | Chile | 0.38 |
| 19 | QUP2425_2009 | MILAN/PASTOR//DOMO | Chile | 0.28 |
| 13 | QUP2450_2005 | QUI 658_94/3/PRL/SARA//TSI/VEE PT5 | Chile | 0.29 |
| 11 | QUP2464_2007 | SITE//BUC/PVN/3/QUELEN | Chile | 0.36 |
| 10 | QUP2474_2007 | SITE//BUC/PVN/3/QUELEN | Chile | 0.29 |
| 20 | QUP2502_2009 | CNO79*2/HE//PRT/ORZ’S’/3/QUI 836_94 | Chile | 0.30 |
| 23 | QUP2503_2009 | CNO79*2/HE//PRT/ORZ’S’/3/QUI 836_94 | Chile | 0.22 |
| 26 | QUP2504_2009 | CNO79*2/HE1//PRT/ORZ’S’/3/QUI 836_94 | Chile | 0.29 |
| 25 | QUP2505_2009 | CNO79*2/HE1//PRT/ORZ’S’/3/QUI 836_94 | Chile | 0.26 |
| 54 | QUP2519_2009 | HD2206_HORK//BUC/BUL/3/QUP 1619_95 | Chile | 0.31 |
| 52 | QUP2521_2009 | HD2206_HORK//BUC/BUL/3/QUP 1619_95 | Chile | 0.27 |
| 53 | QUP2522_2009 | HD2206_HORK//BUC/BUL/3/QUP 1619_95 | Chile | 0.35 |
| 35 | QUP2529_2009 | RL6043/4*NAC//QUP 1861_96 | Chile | 0.33 |
| 37 | QUP2532_2009 | PRINIA/WEAVER//STAR/5/IRENA/CETTIA/5/ND/VE9144//KAL/BB/3/YACO/4/CHIL | Chile | 0.33 |
| 55 | QUP2534_2009 | TAMOI/QUP 1619_95 | Chile | 0.27 |
| 21 | QUP2537_2009 | QUP 1861_96/TEMU 1706_99 | Chile | 0.52 |
| 22 | QUP2538_2009 | QUP 1861_96/TEMU 1706_99 | Chile | 0.34 |
| 32 | QUP2539_2009 | QUP 1861_96/TEMU 1706_99 | Chile | 0.42 |
| 27 | QUP2541_2009 | SKAUZ/2*STAR//QUP 2007_99 | Chile | 0.20 |
| 7 | QUP2542_2004 | QUI 685_94/QUP 1707_95 | Chile | 0.35 |
| 51 | QUP2542_2009 | SKAUZ/2*STAR//QUP 2007_99 | Chile | 0.41 |
| 33 | QUP2543_2009 | SKAUZ/2*STAR//QUP 2007_99 | Chile | 0.20 |
| 34 | QUP2544_2009 | SKAUZ/2*STAR//QUP 2007_99 | Chile | 0.54 |
| 24 | QUP2545_2009 | SKAUZ/2*STAR//QUP 2007_99 | Chile | 0.17 |
| 28 | QUP2546_2009 | MILAN/PASTOR//DOMO | Chile | 0.34 |
| 29 | QUP2547_2009 | MILAN/PASTOR//DOMO | Chile | 0.40 |
| 39 | QUP2553_2009 | MILAN/PASTOR//HUAYUN | Chile | 0.30 |
| 36 | QUP2569_2009 | MILAN/PASTOR//DOMO | Chile | 0.27 |
| 31 | QUP2572_2009 | MILAN/PASTOR//DOMO | Chile | 0.33 |
| 38 | QUP2573_2009 | TEMU 1674_99/TEMU 1578_01 | Chile | 0.27 |
| 6 | QUP2612_2003 | MILAN/QUELEN | Chile | 0.33 |
| 30 | QUP2616_2009 | PFAU/WEAVER*2//PAVON 7S3, + LR47 | Chile | 0.30 |
| 14 | QUP2725_2008 | PASTOR/DOMO | Chile | 0.29 |
| 40 | QUP2759_2008 | R37/GHL121//KSL/BB/3/BUC’S’/BUL’S’/4/CRUZ ALTA/CMH82.493 | Chile | 0.29 |
| 41 | QUP2761_2009 | R37/GHL121//KSL/BB/3/BUC’S’/BUL’S’/4/CRUZ ALTA/CMH82.493 | Chile | 0.29 |
| 42 | QUP2763_2009 | R37/GHL121//KSL/BB/3/BUC’S’/BUL’S’/4/CRUZ ALTA/CMH82.493 | Chile | 0.34 |
| 43 | QUP2775_2009 | PATO/ON//MAYA’S’/3/BB/RON//MAYA’S’/4/MILLALEU//76.1084/FLK’S’/5/DOVE’S’//KLAT/SOREN | Chile | 0.26 |
| 15 | QUP2776_2008 | BUC/FLK//MYNA/VUL/3/PF8619/K342 | Chile | 0.23 |
| 44 | QUP2784_2009 | BUC/FLK//MYNA/VUL/3/DOVE’S’//KLAT/SOREN | Chile | 0.34 |
| 16 | QUP2785_2008 | BUC/FLK//MYNA/VUL/3/DOVE’S’//KLAT/SOREN | Chile | 0.26 |
| 45 | QUP2788_2008 | BUC/FLK//MYNA/VUL/3/DOVE’S’//KLAT/SOREN | Chile | 0.35 |
| 46 | QUP2790_2008 | HUAYUN/4/IOC831/3/CAR853/COC//VEE’S’ | Chile | 0.31 |
| 17 | QUP2793_2008 | HUAYUN/4/IOC831/3/CAR853/COC//VEE’S’ | Chile | 0.23 |
| 56 | FONTAGRO1 | TACUPETO F2001 | Chile | 0.37 |
| 57 | FONTAGRO2 | VOROBEY | Chile | 0.30 |
| 58 | FONTAGRO3 | WEEBILL1 | Chile | 0.38 |
| 59 | FONTAGRO4 | SERI*3//RL6010/4*YR/3/PASTOR/4/BAV92 | Chile | 0.45 |
| 60 | FONTAGRO5 | TOB/ERA//TOB/CNO67/3/PLO/4/VEE#5/5/KAUZ/6/FRET2 | Chile | 0.28 |
| 61 | FONTAGRO6 | FRAME//MILAN/KAUZ/3/PASTOR | Chile | 0.27 |
| 62 | FONTAGRO7 | PASTOR/3/URES/JUN//KAUZ/4/WBLL1 | Chile | 0.32 |
| 63 | FONTAGRO8 | KA/NAC//SERI/RAYON | Chile | 0.60 |
| 64 | FONTAGRO9 | KA/NAC//SERI/RAYON | Chile | 0.30 |
| 65 | FONTAGRO10 | PASTOR/MILAN//MILAN/SHA7 | Chile | 0.39 |
| 66 | FONTAGRO11 | JARU//SHA4/CHIL | Chile | 0.35 |
| 67 | FONTAGRO12 | OASIS/5*BORL95/5/CNDO/R143//ENTE/MEXI75/3/AE.SQ/4/2*OCI | Chile | 0.31 |
| 68 | FONTAGRO13 | CROC_1/AE.SQUARROSA (205)//KAUZ/3/ENEIDA/4/PSN/BOW//MILAN | Chile | 0.29 |
| 69 | FONTAGRO14 | FILIN/IRENA/5/CNDO/R143//ENTE/MEXI_2/3/AEGILOPS SQUARROSA (TAUS)/4/WEAVER/6/PFAU/BOW//VEE#9/3/DUCULA | Chile | 0.23 |
| 70 | FONTAGRO15 | FILIN/IRENA/5/CNDO/R143//ENTE/MEXI_2/3/AEGILOPS SQUARROSA (TAUS)/4/WEAVER/6/PFAU/BOW//VEE#9/3/DUCULA | Chile | 0.39 |
| 71 | FONTAGRO16 | BABAX/LR42//BABAX/3/ER2000 | Chile | 0.31 |
| 72 | FONTAGRO17 | PFAU/MILAN//KA/NAC/3/WBLL1 | Chile | 0.35 |
| 73 | FONTAGRO18 | KS82142/2*WBLL1 | Chile | 0.32 |
| 74 | FONTAGRO19 | CHWL86/6/FILIN/IRENA/5/CNDO/R143//ENTE/MEXI_2/3/AEGILOPS SQUARROSA (TAUS)/4/WEAVER | Chile | 0.35 |
| 75 | FONTAGRO20 | KOH92/WBLL1 | Chile | 0.34 |
| 76 | FONTAGRO21 | SLVS//ATTILA*2/M10 (MUTATED C_306) | Chile | 0.26 |
| 77 | FONTAGRO22 | SLVS//ATTILA*2/M10 (MUTATED C_306) | Chile | 0.30 |
| 78 | FONTAGRO23 | MILAN/BERKUT | Chile | 0.28 |
| 79 | FONTAGRO24 | PYN/BAU//FRET2/3/FRET2 | Chile | 0.42 |
| 80 | FONTAGRO25 | KS82W418/SPN/3/CHEN/AE.SQ//2*OPATA/4/FRET2 | Chile | 0.32 |
| 81 | FONTAGRO26 | KS82W418/SPN/3/CHEN/AE.SQ//2*OPATA/4/FRET2 | Chile | 0.40 |
| 82 | FONTAGRO27 | TMP64/TWN//SDY*2/3/WNG,US/4/FRET2/5/FRET2 | Chile | 0.27 |
| 83 | FONTAGRO28 | TAM200/PRL//PASTOR/4/PASTOR//SITE/MO/3/CHEN/AEGILOPS SQUARROSA (TAUS)//BCN | Chile | 0.27 |
| 84 | FONTAGRO29 | PFAU/MILAN//FUNG MAI 24 | Chile | 0.31 |
| 85 | FONTAGRO30 | CROC_1/AE.SQUARROSA (224)//OPATA/3/WBLL1 | Chile | 0.23 |
| 86 | FONTAGRO31 | PASTOR//HXL7573/2*BAU/3/WBLL1 | Chile | 0.34 |
| 87 | FONTAGRO32 | MEX94.2.19//SOKOLL/WBLL1 | Chile | 0.44 |
| 88 | FONTAGRO33 | MEX94.27.1.20/3/SOKOLL//ATTILA/3*BCN | Chile | 0.29 |
| 89 | FONTAGRO34 | CROC_1/AE.SQUARROSA (205)//KAUZ/3/ATTILA/4/VERDIN | Chile | 0.27 |
| 90 | FONTAGRO35 | MUNIA/CHTO/3/PFAU/BOW//VEE#9/4/CHEN/AEGILOPS SQUARROSA (TAUS)//BCN/5/PBW343//CAR422/ANA | Chile | 0.27 |
| 91 | FONTAGRO36 | NIKNEJAD/TILHI//WBLL1 | Chile | 0.25 |
| 92 | FONTAGRO37 | TNMU/MUNIA//MIRIAM 41 | Chile | 0.41 |
| 93 | FONTAGRO38 | KAMB2/PANDION | Chile | 0.26 |
| 94 | FONTAGRO39 | BOW/URES//KEA/3/SITE/4/HEILO | Chile | 0.38 |
| 95 | FONTAGRO40 | PASTOR/FINSI//HEILO | Chile | 0.35 |
| 96 | FONTAGRO41 | MILAN/MUNIA/3/PASTOR//MUNIA/ALTAR 84/4/MILAN/DUCULA | Chile | 0.28 |
| 97 | FONTAGRO42 | MILAN/MUNIA/3/PASTOR//MUNIA/ALTAR 84/4/MILAN/DUCULA | Chile | 0.33 |
| 98 | FONTAGRO43 | IAN9/JARU/7/TNMU/6/CEP80111/CEP81165/5/MRNG/4/YKT406/3/AG/ASN//ATR | Chile | 0.24 |
| 99 | FONTAGRO44 | CROC_1/AE.SQUARROSA (205)//KAUZ/3/FINSI/4/BABAX/KS93U76//BABAX | Chile | 0.24 |
| 100 | FONTAGRO45 | PANDION/3/BABAX/KS93U76//BABAX | Chile | 0.35 |
| 101 | FONTAGRO46 | PANDION/BERKUT | Chile | 0.42 |
| 102 | FONTAGRO47 | PANDION/BERKUT | Chile | 0.47 |
| 103 | FONTAGRO48 | BABAX/PASTOR//AMAD/3/PRL/2*PASTOR | Chile | 0.32 |
| 104 | FONTAGRO49 | PANDION/BERKUT | Chile | 0.35 |
| 105 | FONTAGRO50 | KLES/FRET2//BERKUT | Chile | 0.36 |
| 106 | FONTAGRO51 | TX81V6614/FINSI/3/BABAX/PASTOR//AMAD | Chile | 0.39 |
| 107 | FONTAGRO52 | TX81V6614/FINSI/3/BABAX/PASTOR//AMAD | Chile | 0.41 |
| 108 | FONTAGRO53 | PFAU/BOW//VEE#9/3/BERKUT | Chile | 0.31 |
| 109 | FONTAGRO54 | PFAU/BOW//VEE#9/3/BERKUT | Chile | 0.30 |
| 110 | FONTAGRO55 | PRINIA/SUNCO | Chile | 0.40 |
| 111 | FONTAGRO56 | TUI//2*SUNCO/SA1166/3/TUI/4/METSO/5/URES/PRL//BAV92 | Chile | 0.32 |
| 112 | FONTAGRO57 | PSN/BOW//MILAN/3/WBLL1 | Chile | 0.22 |
| 113 | FONTAGRO58 | MILAN/DUCULA//BERKUT | Chile | 0.30 |
| 114 | FONTAGRO59 | VEE/LIRA//BOW/3/BCN/4/KAUZ/5/WBLL1/6/FINSI | Chile | 0.18 |
| 115 | FONTAGRO60 | SOKOLL/SUNVALE | Chile | 0.26 |
| 116 | FONTAGRO61 | VEE/MJI//2*TUI/3/2*PASTOR/4/BERKUT/5/PFAU/MILAN | Chile | 0.28 |
| 117 | FONTAGRO62 | VEE/MJI//2*TUI/3/2*PASTOR/4/BERKUT/5/PFAU/MILAN | Chile | 0.31 |
| 118 | FONTAGRO63 | PRINIA/BERKUT//PFAU/MILAN | Chile | 0.43 |
| 119 | FONTAGRO64 | NG8675/CBRD//MILAN/3/BERKUT/4/SOKOLL | Chile | 0.25 |
| 120 | FONTAGRO65 | JNRB.2/3/BABAX/PASTOR//AMAD | Chile | 0.23 |
| 121 | FONTAGRO66 | FRET2//SKAUZ*2/FCT/3/FILIN/2*PASTOR | Chile | 0.29 |
| 122 | FONTAGRO67 | SHAM4//VEE#5/NAC/3/ATTILA*2/M10 (MUTATED C_306)/4/WBLL1 | Chile | 0.41 |
| 123 | FONTAGRO68 | BABAX/LR42//BABAX/3/HXL7573/2*BAU/10/ATTILA*2/9/KT/BAGE//FN/U/3/BZA/4/TRM/5/ALDAN/6/SERI/7/VEE#10/8/OPATA | Chile | 0.35 |
| 124 | FONTAGRO69 | PSN/BOW//MILAN/3/PRL/2*PASTOR | Chile | 0.23 |
| 125 | FONTAGRO70 | MILAN//SRMA/TUI/3/PRL/2*PASTOR | Chile | 0.31 |
| 126 | FONTAGRO71 | KLDR/PEWIT1//MILAN/DUCULA | Chile | 0.30 |
| 127 | FONTAGRO72 | SW89_5124*2/FASAN/6/CHIBIA/5/CNDO/R143//ENTE/MEXI_2/3/AEGILOPS SQUARROSA (TAUS)/4/WEAVER | Chile | 0.27 |
| 128 | FONTAGRO73 | SUN371A/3/CHEN/AE.SQ//WEAVER | Chile | 0.45 |
| 129 | FONTAGRO74 | EXCALIBUR//ALTAR 84/AE.SQUARROSA (502)/3/SUNCO | Chile | 0.31 |
| 130 | FONTAGRO75 | T.TAU.83.2.36//SUNCO/2*PASTOR | Chile | 0.23 |
| 131 | FONTAGRO76 | SOKOLL/EXCALIBUR | Chile | 0.32 |
| 132 | FONTAGRO77 | SOKOLL/EXCALIBUR | Chile | 0.39 |
| 133 | FONTAGRO78 | BAXTER*2/4/CHEN/AEGILOPS SQUARROSA (TAUS)//BCN/3/BAV92 | Chile | 0.33 |
| 134 | FONTAGRO79 | T.DICOCCON PI94625/AE.SQUARROSA (372)//3*PASTOR/3/PANDORA | CIMMYT | 0.44 |
| 135 | FONTAGRO80 | T.DICOCCON PI94625/AE.SQUARROSA (372)//3*PASTOR/3/PANDORA | CIMMYT | 0.36 |
| 136 | FONTAGRO81 | ITP45*2/BERKUT | CIMMYT | 0.19 |
| 137 | FONTAGRO82 | DUMA/4/TC14/2*HTG//DUCULA/3/PRINIA | CIMMYT | 0.32 |
| 138 | FONTAGRO83 | PANDORA*2/3/FRET2/KUKUNA//FRET2 | CIMMYT | 0.28 |
| 139 | FONTAGRO84 | PANDORA*2/3/FRET2/KUKUNA//FRET2 | CIMMYT | 0.36 |
| 140 | FONTAGRO85 | PANDORA*2/3/FRET2/KUKUNA//FRET2 | CIMMYT | 0.36 |
| 141 | FONTAGRO86 | CROC_1/AE.SQUARROSA (224)//OPATA/4/TC14/2*HTG//DUCULA/3/PRINIA | CIMMYT | 0.24 |
| 142 | FONTAGRO87 | PANDORA*2/3/FRET2/KUKUNA//FRET2 | CIMMYT | 0.33 |
| 143 | FONTAGRO88 | PANDORA*2/3/FRET2/KUKUNA//FRET2 | CIMMYT | 0.22 |
| 144 | FONTAGRO89 | PANDORA*2/3/FRET2/KUKUNA//FRET2 | CIMMYT | 0.33 |
| 145 | FONTAGRO90 | PALOMAR/4/BJY/COC//PRL/BOW/3/FRTL | CIMMYT | 0.44 |
| 146 | FONTAGRO91 | CUNNINGHAM/BERKUT | CIMMYT | 0.32 |
| 147 | FONTAGRO92 | PFAU/BOW//VEE#9/3/WBLL1 | CIMMYT | 0.47 |
| 148 | FONTAGRO93 | SUNVALE/PASTOR/3/BABAX/PASTOR//AMAD | CIMMYT | 0.41 |
| 149 | FONTAGRO94 | SW94.2690/BERKUT/10/ATTILA*2/9/KT/BAGE//FN/U/3/BZA/4/TRM/5/ALDAN/6/SERI/7/VEE#10/8/OPATA | CIMMYT | 0.38 |
| 150 | FONTAGRO95 | MILAN/DUCULA//BERKUT | CIMMYT | 0.39 |
| 151 | FONTAGRO96 | FILIN/IRENA/5/CNDO/R143//ENTE/MEXI_2/3/AEGILOPS SQUARROSA (TAUS)/4/WEAVER/6/BERKUT | CIMMYT | 0.30 |
| 152 | FONTAGRO97 | T.TAU.83.2.36/BERKUT | CIMMYT | 0.29 |
| 153 | FONTAGRO98 | FILIN/IRENA/5/CNDO/R143//ENTE/MEXI_2/3/AEGILOPS SQUARROSA (TAUS)/4/WEAVER/6/BERKUT | CIMMYT | 0.18 |
| 154 | FONTAGRO99 | SUN371A*2/3/CHEN/AE.SQ//WEAVER | CIMMYT | 0.30 |
| 155 | FONTAGRO100 | SOKOLL/EXCALIBUR | CIMMYT | 0.36 |
| 156 | FONTAGRO101 | SOKOLL/EXCALIBUR | CIMMYT | 0.31 |
| 157 | FONTAGRO102 | SOKOLL/EXCALIBUR | CIMMYT | 0.24 |
| 158 | FONTAGRO103 | SOKOLL/EXCALIBUR | CIMMYT | 0.31 |
| 159 | FONTAGRO104 | SOKOLL/EXCALIBUR | CIMMYT | 0.27 |
| 160 | FONTAGRO105 | MILAN/DUCULA//SUNCO/2*PASTOR | CIMMYT | 0.19 |
| 161 | FONTAGRO106 | SOKOLL/KENNEDY//LANG | CIMMYT | 0.20 |
| 162 | FONTAGRO107 | CHIBIA/5/CNDO/R143//ENTE/MEXI_2/3/AEGILOPS SQUARROSA (TAUS)/4/WEAVER/6/DULUS | CIMMYT | 0.25 |
| 163 | FONTAGRO108 | CHIBIA/5/CNDO/R143//ENTE/MEXI_2/3/AEGILOPS SQUARROSA (TAUS)/4/WEAVER/6/DULUS | CIMMYT | 0.33 |
| 164 | FONTAGRO109 | SUNCO/2*PASTOR/4/CROC_1/AE.SQUARROSA (224)//YACO/3/MUNIA | CIMMYT | 0.43 |
| 165 | FONTAGRO110 | SOKOLL/KENNEDY//LANG | CIMMYT | 0.14 |
| 166 | FONTAGRO111 | KONK/4/CROC_1/AE.SQUARROSA (205)//KAUZ/3/ATTILA | CIMMYT | 0.30 |
| 167 | FONTAGRO112 | NG8675/CBRD//MILAN/3/BERKUT/4/PFAU/BOW//VEE#9/3/DUCULA | CIMMYT | 0.26 |
| 168 | FONTAGRO113 | MTRWA92.161/PRINIA/5/SERI*3//RL6010/4*YR/3/PASTOR/4/BAV92 | CIMMYT | 0.46 |
| 169 | FONTAGRO114 | MTRWA92.161/PRINIA/5/SERI*3//RL6010/4*YR/3/PASTOR/4/BAV92 | CIMMYT | 0.32 |
| 170 | FONTAGRO115 | D67.2/PARANA 66.270//AE.SQUARROSA (220)/3/2*BERKUT | CIMMYT | 0.39 |
| 171 | FONTAGRO116 | QT8368/BERKUT//SUNCO/2*PASTOR | CIMMYT | 0.25 |
| 172 | FONTAGRO117 | SOKOLL//PRL/2*PASTOR | CIMMYT | 0.29 |
| 173 | FONTAGRO118 | BERKUT/HTG | CIMMYT | 0.28 |
| 174 | FONTAGRO119 | CROC_1/AE.SQUARROSA (205)//BORL95/3/KENNEDY/6/CNDO/R143//ENTE/MEXI_2/3/AEGILOPS SQUARROSA (TAUS)/4/WEAVER/5/2*JANZ | CIMMYT | 0.25 |
| 175 | FONTAGRO120 | ASTREB*2/NING MAI 9558 | CIMMYT | 0.41 |
| 176 | FONTAGRO121 | ASTREB*2/CBRD | CIMMYT | 0.28 |
| 177 | FONTAGRO122 | ATTILA/3/URES/PRL//BAV92/4/WBLL1 | CIMMYT | 0.43 |
| 178 | FONTAGRO123 | 92.001E7.32.5/SLVS | CIMMYT | 0.29 |
| 179 | FONTAGRO124 | KS82W418/SPN/3/CHEN/AE.SQ//2*OPATA/4/FRET2 | CIMMYT | 0.23 |
| 180 | FONTAGRO125 | FOW 1 | Chile | 0.32 |
| 181 | FONTAGRO126 | BABAX/LR42//BABAX/3/ER2000 | Chile | 0.24 |
| 182 | FONTAGRO127 | TC870344/GUI//TEMPORALERA M 87/AGR/3/2*WBLL1 | Chile | 0.29 |
| 183 | FONTAGRO128 | KS82142/2*WBLL1 | Chile | 0.34 |
| 184 | FONTAGRO129 | SLVS//ATTILA*2/M10 (MUTATED C_306) | Chile | 0.36 |
| 185 | FONTAGRO130 | MILAN//PRL/2*PASTOR | Chile | 0.26 |
| 186 | FONTAGRO131 | T. DICOCCON PI94625/AE,SQUARROSA (372)//3*PASTOR | Chile | 0.35 |
| 187 | FONTAGRO132 | PASTOR/4/WEAVER/TSC//WEAVER/3/WEAVER/5/URES/PRL//BAV92 | Chile | 0.33 |
| 188 | FONTAGRO133 | SW94,2690/SUNCO | Chile | 0.44 |
| 189 | FONTAGRO134 | SW94,2690/SUNCO | Chile | 0.42 |
| 190 | FONTAGRO135 | KS82W418/SPN/3/CHEN/AE.SQ//2*OPATA/4/FRET2 | Chile | 0.30 |
| 191 | FONTAGRO136 | TAN//TEMPORALERA M 87/AGR/3/FRET2/4/URES/PRL//BAV92 | Chile | 0.29 |
| 192 | FONTAGRO137 | PFAU/MILAN//FUNG MAI 24 | Chile | 0.38 |
| 193 | FONTAGRO138 | PFAU/MILAN//FUNG MAI 24 | Chile | 0.29 |
| 194 | FONTAGRO139 | SOKOLL/WBLL1 | Chile | 0.35 |
| 195 | FONTAGRO140 | SOKOLL/WBLL1 | Chile | 0.28 |
| 196 | FONTAGRO141 | SOKOLL/WBLL1 | Chile | 0.32 |
| 197 | FONTAGRO142 | SOKOLL/WBLL1 | Chile | 0.28 |
| 198 | FONTAGRO143 | MEX94,2,19//SOKOLL/WBLL1 | Chile | 0.31 |
| 199 | ORL99192 |  | Uruguay | 0.18 |
| 200 | SilviaG1 | SUZ6/OPATA | Uruguay | 0.25 |
| 201 | Parula | FKN/3/2*FCR//KAD/GB54/4/BB/CHA | Uruguay | 0.33 |
| 202 | Toropi | FRONTANA/QUADERNA A//PETIBLANCO 8 | Uruguay | 0.18 |
| 203 | SilviaG2 | GENARO*3/PARULA | Uruguay | 0.46 |
| 204 | OCORONI | GRAJO//EMU/JUPATECO_F_73 | CIMMYT | 0.39 |
| 205 | Tonichi |  | CIMMYT | 0.29 |
| 206 | SilviaG3 | BAU/KAUZ | CIMMYT | 0.31 |
| 207 | SAAR | SNI/TRAP#1//BAV92 | CIMMYT | 0.31 |
| 208 | CHAPIO | CARIANCA_422/ANAHUAC_F_75//YACO/3/KAUZ*2/TRAP//KAUZ | CIMMYT | 0.15 |
| 209 | Tukuru | TRAP#1/YACO/3/KAUZ*2/TRAP//KAUZ | CIMMYT | 0.17 |
| 210 | Amadina | BOBWHITE/CROW//BUCK//PAVONF76/3/VEER#10 | CIMMYT | 0.20 |
| 211 | SilviaG4 | BR23//CEP19/PF854 90 | Uruguay | 0.23 |
| 212 | SilviaG5 | CEP 8749/BR 35 | Uruguay | 0.23 |
| 213 | SilviaG6 | BPON/PBRED/4/R37/GHL21//KAL/BB/3/KL T.S | Uruguay | 0.33 |
| 214 | SilviaG7 | I.TORCAZA*2//CEP8749/EMBRAPA_27 | Uruguay | 0.29 |
| 215 | SilviaG8 | LE 2304*2/PARULA | Uruguay | 0.31 |
| 216 | SilviaG9 | LE 2304*2/ PARULA | Uruguay | 0.23 |
| 217 | SilviaG10 | GENARO*3/PARULA//LE2252 | Uruguay | 0.33 |
| 218 | SilviaG11 | LE 2304*2//CEP8749/EMBRAPA_27 | Uruguay | 0.29 |
| 219 | SilviaG12 | LE 2304*2//CEP8749/EMBRAPA_27 | Uruguay | 0.22 |
| 220 | SilviaG13 | I.TIJERETA*2/SUZ6/OPATA | Uruguay | 0.30 |
| 221 | SilviaG14 | I.TIJERETA*2/TORO PI | Uruguay | 0.25 |
| 222 | SilviaG15 | PARULA/ORL 99192*2 | Uruguay | 0.29 |
| 223 | SilviaG16 | I.GORRION*2/ CHAPIO | Uruguay | 0.19 |
| 224 | LE2263_CATBIRD | BAGULA//CHUAN_MAI_18 | BAGULA | 0.30 |
| 225 | LE2283 | BOW//BUC/BUL | Uruguay | 0.32 |
| 226 | LE2279 | BCHA/4/JUP/3/7C/PATO//LR64 | Uruguay | 0.30 |
| 227 | LE2265 | PGO//CHEN/AE.SQUARROSA(224)/3/WEAVER | Uruguay | 0.44 |
| 228 | LE2305 | BCHA/MILAN | Uruguay | 0.33 |
| 229 | LE2318 | IBOY//TRAP#1/BOW | Uruguay | 0.26 |
| 230 | Boyero | LE 2172 | Uruguay | 0.24 |
| 231 | LE2319 | ICAB/ITIJ | Uruguay | 0.23 |
| 232 | LE2352 | MN92045//SUZ3/VEE5 | Uruguay | 0.45 |
| 233 | LE2357 | MN93346/CBRD | Uruguay | 0.33 |
| 234 | LE2367 | LE2265/LE2304 | Uruguay | 0.25 |
| 235 | LE2368 | KDENR/LE2302 | Uruguay | 0.26 |
| 236 | MarthaD1 | KABY//2*ALUBUC/BAYA/3/2*PIGR | Uruguay | 0.25 |
| 237 | MarthaD2 | KABY//2*ALUBUC/BAYA/3/2*PIGR | Uruguay | 0.43 |
| 238 | MarthaD3 | ALTAR84/AEGILOPS SQUARROSA(TAUS)//OCI/3/VEE/MJI//2*TUI/4/2*PIGR | Uruguay | 0.37 |
| 239 | MarthaD4 | ALTAR84/AEGILOPS SQUARROSA(TAUS)//OCI/3/VEE/MJI//2*TUI/4/2*PIGR | Uruguay | 0.37 |
| 240 | MarthaD5 | KRONSTAD F2004/2*INIA TERO | Uruguay | 0.25 |
| 241 | MarthaD6 | KRONSTAD F2004/2*INIA TERO | Uruguay | 0.21 |
| 242 | MarthaD7 | KRONSTAD F2004/2*INIA TERO | Uruguay | 0.22 |
| 243 | MarthaD8 | KABY // 2*ALUBUC/BAYA/3/2*INIA TERO | Uruguay | 0.39 |
| 244 | MarthaD9 | KRONSTAD F2004/2*ITP45 | Uruguay | 0.25 |
| 245 | MarthaD10 | KRONSTAD F2004/2*ITP45 | Uruguay | 0.32 |
| 246 | MarthaD11 | KRONSTAD F2004/2*ITP45 | Uruguay | 0.32 |
| 247 | MarthaD12 | ALTAR 84/AEGILOPS SQUARROSA (TAUS)//OCI/3/VEE/MJI//2*TUI/4/2*ITP45 | Uruguay | 0.34 |
| 248 | MarthaD13 | ALTAR 84/AEGILOPS SQUARROSA (TAUS)//OCI/3/VEE/MJI//2*TUI/4/2*ITP45 | Uruguay | 0.18 |
| 249 | MarthaD14 | ALSEN/7/RFN*2//908/FN/3/MD/4/KKZ/5/BR23/6/CEP 8466 (LACOS 14_241) | Uruguay | 0.15 |
| 250 | MarthaD15 | ALSEN/7/RFN*2//908/FN/3/MD/4/KKZ/5/BR23/6/CEP 8466 (LACOS 14_241) | Uruguay | 0.16 |
| 251 | MarthaD16 | I.TORCAZA/FRONTANA | Uruguay | 0.17 |
| 252 | Condor |  | Uruguay | 0.36 |
| 253 | LE2350 | ICAB/LE2242 | Uruguay | 0.28 |
| 254 | LE2362 | LE2233/COKER6_88 | Uruguay | 0.23 |
| 255 | LE2366 | U1294_9_2_2_1/U1275_1_4_2//ITIJ | Uruguay | 0.21 |
| 256 | LE2369 | LE2233/COKER6_88 | Uruguay | 0.22 |
| 257 | LE2373 | TNMU/3/EMB27/CEP8825//MILAN | Uruguay | 0.20 |
| 258 | LE2375 | LET0113_31_7E_0E_0E_3E_0E | Uruguay | 0.27 |
| 259 | LE2376 | LE2304/BAG10 | Uruguay | 0.25 |
| 260 | LE2381 | BAG10/B. Arriero | Uruguay | 0.33 |
| 261 | LE2382 | LE2265/3/PF9099/OR1//GRANITO | Uruguay | 0.35 |
| 262 | LE2383 | PF9099/OR1//GRANITO/3/BAG10 | Uruguay | 0.31 |
| 263 | LE2384 | BAG10/B. Sureño | Uruguay | 0.48 |
| 264 | LE2386 | LE2305/BAG10 | Uruguay | 0.33 |
| 265 | LE2387 | PF9099/OR1//GRANITO/3/BAG10 | Uruguay | 0.36 |
| 266 | LE2388 | BABAX/LR42//BABAX*2/3/KURUKU | Uruguay | 0.41 |
| 267 | LE2389 | SHAAN 229/3/SHA3/SERI//G.C.W 1/SERI | Uruguay | 0.26 |
| 268 | LE2390 | LE2305/3/PF9099/OR1//GRANITO | Uruguay | 0.20 |
| 269 | Cardenal | Mengovi/8156//JoralSib/SieteCerros66 | Uruguay | 0.33 |
| 270 | Efederal | EHOR/CNT8 | Uruguay | 0.32 |
| 271 | Epelon90 | KVZ/TRM | Uruguay | 0.33 |
| 272 | Mirlo | CAR853//COC/VEE5/3/URES | Uruguay | 0.25 |
| 273 | INIA_TIJERETA | LE2132/ECAL | Uruguay | 0.29 |
| 274 | Gorrion_LE2245 | EFED/ECOL | Uruguay | 0.28 |
| 275 | Churrinche | EFED/LE2154 | Uruguay | 0.21 |
| 276 | Tero | LI107/C_CH_91_1642 | Uruguay | 0.38 |
| 277 | Carancho | EFED/LE2187 | Uruguay | 0.33 |
| 278 | DonAlberto_LE2331 | ITIJ/LE2229 | Uruguay | 0.47 |
| 279 | Carpintero_LE2333 | ECAR/CATBIRD'S | Uruguay | 0.29 |
| 280 | Madrugador_LE2332 | EFED//CHUANMAI/BAU | Uruguay | 0.22 |
| 281 | Genesis2354 | LE2252/LE2265 | Uruguay | 0.19 |
| 282 | F6_CL_09_13183 | LE2304*2/PARULA | Uruguay | 0.22 |
| 283 | RESEL_CL_09_813 | LE2265/PELI | Uruguay | 0.39 |
| 284 | F6_CL_09_843 | LE2299/ALSEN | Uruguay | 0.19 |
| 285 | F6_CL_09_1049 | LE2301/LE2319 | Uruguay | 0.44 |
| 286 | F6_CCCI_DSU_09_15274 | LE2314/ALSEN | Uruguay | 0.31 |
| 287 | F6_CCCI_DSU_09_15280 | LE2314/ALSEN | Uruguay | 0.24 |
| 288 | F6_CL_09_1382 | LE2304*2/PARULA | Uruguay | 0.20 |
| 289 | F6_CCCI_DSU_09_15302 | LE2316/ALSEN | Uruguay | 0.26 |
| 290 | F6_CCCI_09_13819 | ICHU*2/TUKURU | Uruguay | 0.20 |
| 291 | F6_CCCI_09_1619 | ICHU/ALSEN | Uruguay | 0.41 |
| 292 | F6_CCCI_09_1751 | IGAR/ICHU | Uruguay | 0.39 |
| 293 | F6_CCCI_09_1900 | IGORR/ALSEN | Uruguay | 0.25 |
| 294 | F6_CCCI_09_1943 | ITER/LE2265 | Uruguay | 0.29 |
| 295 | F6_CCCI_09_2258 | LE2304//SHANGAI#5/WEAVER | Uruguay | 0.31 |
| 296 | F6_CCCI_09_2374 | LE2312/ALSEN | Uruguay | 0.26 |
| 297 | F6_CCCI_09_2379 | LE2312/BAG10 | Uruguay | 0.27 |
| 298 | F6_CCCI_09_2378 | LE2312/BAG10 | Uruguay | 0.20 |
| 299 | F6_CCCI_09_2589 | LE2316/LE2318 | Uruguay | 0.20 |
| 300 | F6_CCCI_09_13362 | ICON*2/SAAR | Uruguay | 0.27 |
| 301 | F6_CCCI_09_13450 | ITOR*2//CEP8749/EMBRAPA27 | Uruguay | 0.24 |
| 302 | F6_CCCI_09_13636 | LE2304*2/PARULA | Uruguay | 0.28 |
| 303 | F6_CCCI_09_13377 | PARULA/ORL99192*2 | Uruguay | 0.33 |
| 304 | F6_CL_DSU_09_10005 | BAG10/2/EFED/W92_7351 | Uruguay | 0.25 |
| 305 | F7_09_13234 | Genaro*3/Parula//LE2252 | Uruguay | 0.28 |
| 306 | F6_CCCI_09_1561 | ICARA/ALSEN | Uruguay | 0.30 |
| 307 | F6_09_13893 | ICHU*2/TUKURU | Uruguay | 0.22 |
| 308 | F6_CCCI_09_1589 | ICHU/5/CEP85155/3/CEP7780*2//H499.71A/4*JUP/4/BR23 | Uruguay | 0.29 |
| 309 | F6_CCCI_09_1597 | ICHU/ALSEN | Uruguay | 0.21 |
| 310 | F6_09_13327 | ICON*2/OCORONI | Uruguay | 0.32 |
| 311 | F7_09_13045 | ICON/TUKURU | Uruguay | 0.38 |
| 312 | F6_CCCI_09_1659 | IGAR/4/KLAT/MJI/VI/3/PSN"S"/BOW"S" | Uruguay | 0.25 |
| 313 | F6_CCCI_09_1713 | IGAR/ALSEN | Uruguay | 0.30 |
| 314 | F6_CCCI_09_1724 | IGAR/BAG10 | Uruguay | 0.18 |
| 315 | F6_CCCI_DSU_09_15150 | IGAR/LE2334 | Uruguay | 0.21 |
| 316 | F6_CCCI_DSU_09_15160 | IGAV/3/REMUS//SUMAI3/THOMBIRD | Uruguay | 0.29 |
| 317 | F6_CCCI_09_1832 | IGAV/ALSEN | Uruguay | 0.34 |
| 318 | F6_CCCI_09_1836 | IGAV/LE2283 | Uruguay | 0.38 |
| 319 | RESEL_CL_09_656 | IGORR//TUI/CLMS | Uruguay | 0.37 |
| 320 | F6_CCCI_09_1890 | IGORR/5/CEP85155/3/CEP7780*2//H499.71A/4*JUP/4/BR23 | Uruguay | 0.35 |
| 321 | F6_CCCI_DSU_09_15201 | ITIJ/IMAD | Uruguay | 0.20 |
| 322 | F6_CCCI_DSU_09_15238 | LE2299/ALSEN | Uruguay | 0.22 |
| 323 | F6_CCCI_DSU_09_15258 | LE2301/PGAU | Uruguay | 0.21 |
| 324 | F6_CCCI_09_2203 | LE2302/ALSEN | Uruguay | 0.23 |
| 325 | F6_CCCI_09_2197 | LE2302/ALSEN | Uruguay | 0.19 |
| 326 | F6_CCCI_09_2219 | LE2302/ALSEN | Uruguay | 0.18 |
| 327 | F6_09_13757 |  | Uruguay | 0.23 |
| 328 | F6_CCCI_09_2281 | LE2304/ALSEN | Uruguay | 0.26 |
| 329 | F6_CCCI_09_2333 | LE2309/ALSEN | Uruguay | 0.16 |
| 330 | F6_CCCI_09_2360 | LE2312/4/TRAP1/YACO/3/KAUZ*2/TRAP//KAUZ | Uruguay | 0.33 |
| 331 | F6_CL_09_1337 | LE2314/ALSEN | Uruguay | 0.16 |
| 332 | F6_CCCI_09_2452 | LE2314/ALSEN | Uruguay | 0.20 |
| 333 | F6_CCCI_DSU_09_15279 | LE2314/ALSEN | Uruguay | 0.29 |
| 334 | F6_CCCI_09_2540 | LE2315/ALSEN | Uruguay | 0.32 |
| 335 | F6_CCCI_DSU_09_15301 | LE2315/IMAD | Uruguay | 0.21 |
| 336 | F6_CL_DSU_09_10354 | LE2316/ALSEN | Uruguay | 0.32 |
| 337 | F6_CCCI_09_2636 | LE2317/BAG10 | Uruguay | 0.23 |
| 338 | F6_CCCI_09_2680 | LE2318/ALSEN | Uruguay | 0.24 |
| 339 | F6_CCCI_09_2714 | LE2319/ALSEN | Uruguay | 0.20 |
| 340 | F6_CCCI_DSU_09_15323 | Ringo Sztár_MM/NB//ITIJ/3/LE2302 | Uruguay | 0.26 |
| 341 | F7_CCCI_08_7386 | BAG10//EFED*2/TcLr21,34 | Uruguay | 0.37 |
| 342 | F7_CCCI_08_7388 | BAG10//EFED*2/TcLr21,34 | Uruguay | 0.35 |
| 343 | F6_CCCI_08_14555 | BAG10//IBOY/LE2302 | Uruguay | 0.41 |
| 344 | F6_CCCI_08_14634 | FUNDACEP30/EPEL90 | Uruguay | 0.27 |
| 345 | F6_CCCI_08_15169 | ICARA/BAG10 | Uruguay | 0.27 |
| 346 | F4_CCCI_08_176 | ICARP/3/SUZHOE#10//ALD"S"/PVN"S" | Uruguay | 0.33 |
| 347 | F4_CCCI_08_202 | ICARP/ONIX | Uruguay | 0.37 |
| 348 | F4_CCCI_08_262 | IDALB/ACA302 | Uruguay | 0.43 |
| 349 | F4_CCCI_08_283 | IDALB/ICON | Uruguay | 0.39 |
| 350 | F4_CCCI_08_348 | IGAR/IDALB | Uruguay | 0.31 |
| 351 | F7_CCCI_08_7424 | ITER/LE2265 | Uruguay | 0.34 |
| 352 | F6_CCCI_08_14753 | ITIJ/3/PF9099/OR1//GRANITO | Uruguay | 0.26 |
| 353 | F4_CCCI_08_508 | ITIJ/ONIX | Uruguay | 0.32 |
| 354 | F7_CCCI_08_7431 | LE2265/3/PF9099/OR1//GRANITO | Uruguay | 0.21 |
| 355 | F6_CCCI_08_15204 | LE2265/CEP8386 | Uruguay | 0.30 |
| 356 | F6_CCCI_08_14834 | LE2301/3/PF9099/OR1//GRANITO | Uruguay | 0.26 |
| 357 | F7_CCCI_08_7446 | LE2302/3/PF9099/OR1//GRANITO | Uruguay | 0.25 |
| 358 | F6_CCCI_08_14921 | LE2302/BAG10 | Uruguay | 0.16 |
| 359 | F6_CCCI_08_14935 | LE2305/3/PF9099/OR1//GRANITO | Uruguay | 0.26 |
| 360 | F6_CCCI_08_14961 | LE2305/BAG10 | Uruguay | 0.35 |
| 361 | F3_CCCI_08_17200 | ONIX/PARULA | Uruguay | 0.23 |
| 362 | F6_CCCI_08_14995 | ORL 97150/Catbird | Uruguay | 0.20 |
| 363 | F6_CCCI_08_14996 | ORL 97150/Catbird | Uruguay | 0.19 |
| 364 | F6_CCCI_08_15233 | ORL99192//CATBIRD'S'/IBOY | Uruguay | 0.21 |
| 365 | F6_CCCI_08_15019 | ORL99192//ECAR/CATBIRD'S' | Uruguay | 0.23 |
| 366 | F6_CCCI_08_15023 | ORL99192//ECAR/CATBIRD'S' | Uruguay | 0.25 |
| 367 | F6_CCCI_08_15247 | ORL99192/4/MILAN'S'/3/EFED.//BUCK 6/MR 74507 | Uruguay | 0.28 |
| 368 | F6_CCCI_08_15250 | ORL99192/4/MILAN'S'/3/EFED.//BUCK 6/MR 74507 | Uruguay | 0.23 |
| 369 | F6_CCCI_08_15053 | ORL99192/ITAPUA 50_ AMISTAD | Uruguay | 0.36 |
| 370 | F6_CCCI_08_15078 | PF9099/OR1//GRANITO/3/EFED*2/TcLr21,34 | Uruguay | 0.29 |
| 371 | F6_CL_08_11516 | PF9099/OR1//GRANITO/3/EFED*2/TcLr21,34 | Uruguay | 0.24 |
| 372 | Biointa1001 |  | Uruguay | 0.24 |
| 373 | Onix | CEP 24/RUBI SIB | Uruguay | 0.26 |
| 374 | Nogal |  | Uruguay | 0.32 |
| 375 | Atlax |  | Uruguay | 0.26 |
| 376 | LE2394 | ORL 99192/IGAV | Uruguay | 0.29 |
| 377 | IAPAR_Lacos25_97 |  | Uruguay | 0.23 |
| 378 | Kproteo | KVZ/K4500.L.A.4//VEE"S"/3/KLCBR/4/H1928m | Uruguay | 0.32 |
| 379 | Centauro |  | Uruguay | 0.34 |
| 380 | Cronox | PF9099/OR1//GRANITO | Uruguay | 0.34 |
| 381 | IC03_136 | 494J6.11/RDWG | Uruguay | 0.33 |
| 382 | Kchaja | NANJING/3/BUC"S"//H697/DKL | Uruguay | 0.28 |
| 383 | FUSTROX06_19 | CEP24/PF87107//PVN/ANI´S´ | Uruguay | 0.21 |
| 384 | LE2341 | LE2199/CHIRYA3 | Uruguay | 0.35 |

Table S2. F values of ANOVA for agronomic and physiological traits, for 378 genotypes of wheat grown under severe water stress (Cauquenes WS) and full irrigation (Santa Rosa FI) in two growing seasons.

| Agronomic  traits | Year | Genotype (G) | Environment (E) | G x E | Physiological  traits | Year | Genotype (G) | Environment (E) | G x E |
| --- | --- | --- | --- | --- | --- | --- | --- | --- | --- |
| GY | 2011^1^ | 1.2* | 5001.9*** | 0.6 | SPADa | 2011^1^ | 4.1*** | 211*** | 2.6*** |
|  | 2012 | 2.5*** | 24217*** | 1.8*** |  | 2012 | 4.6*** | 4633*** | 2.0*** |
| DH | 2011 | - | - | - | SPADg | 2011 | - | - | - |
|  | 2012 | - | - | - |  | 2012 | 2.4*** | 1952*** | 1.8*** |
| PH | 2011 | 4.0*** | 12052*** | 1.4** | SWa | 2011 | 0.7 | 76*** | 0.4 |
|  | 2012 | 3.3*** | 543*** | 1.1 |  | 2012 | 5.3*** | 2398*** | 1.3** |
| SM2 | 2011 | 4.2*** | 1310*** | 1.4** | SWg | 2011 | 0.5 | 128*** | 0.4 |
|  | 2012 | 5.5*** | 7978*** | 2.1*** |  | 2012 | 6.2*** | 0.9 | 1.3** |
| KS | 2011 | 6.6*** | 2383*** | 1.8*** | WSCa | 2011 | 1.3* | 133*** | 1.0 |
|  | 2012 | 3.8*** | 613*** | 1.6*** |  | 2012 | 1.2* | 1249*** | 1.2* |
| TKW | 2011 | 15.1*** | 6725*** | 1.4** | WSCg | 2011 | 6.7*** | 2963*** | 6.6*** |
|  | 2012 | 14.1*** | 906*** | 1.3** |  | 2012 | 1.5*** | 13*** | 1.1 |
| KM2 | 2011 | 2.2*** | 1860*** | 0.9 | WSCCa | 2011 | 0.7 | 3.8* | 0.5 |
|  | 2012 | 2.6*** | 6722*** | 1.7*** |  | 2012 | 2.4*** | 2305*** | 1.3** |
|  |  |  |  |  | WSCCg | 2011 | 1.3* | 78*** | 1.1 |
|  |  |  |  |  |  | 2012 | 1.8*** | 7.7** | 1.2** |
|  |  |  |  |  | Δ^13^C | 2011 | 1.1 | 7512*** | 0.7 |
|  |  |  |  |  |  | 2012 | 4.3*** | 45500*** | 1.6*** |

^1^ In 2011 ten genotypes were discarded from the analysis due to low spike numbers. * P< 0.05; ** P< 0.001; *** P< 0.0001
